# Synthesis and biological evaluation of novel quaternary ammonium antibody drug conjugates based on camptothecin derivatives

**DOI:** 10.1371/journal.pone.0292871

**Published:** 2023-12-19

**Authors:** Yifan Zhang, Mengyuan Ding, Lei Wang, Sicheng Yin, Liang Zhang, Xuemei Cao, Zhiyang Chen, Weinan Li, Qingsong Guo, Shulei Zhu, Wei Lu, Tong Yang

**Affiliations:** 1 State Key Laboratory of Genetic Engineering, Department of Biochemistry, School of Life Sciences, Fudan University, Shanghai, China; 2 R&D Department of Genetic Engineering, Shanghai Fudan-Zhangjiang Bio-Pharmaceutical Co., Ltd., Shanghai, China; 3 Shanghai Engineering Research Center of Molecular Therapeutics and New Drug Development, School of Chemistry and Molecular Engineering, East China Normal University, Shanghai, PR China; 4 Innovation Center for AI and Drug Discovery, East China Normal University, Shanghai, PR China; 5 Shanghai Key Laboratory of Intelligent Drug Design and Manufacturing, East China Normal University, Shanghai, PR China; Sichuan University West China Hospital, CHINA

## Abstract

Antibody drug conjugates (ADCs) have emerged as a highly promising class of cancer therapeutics, comprising antibodies, effector molecules, and linkers. Among them, DS-8201a with DXd as the effector molecule, has shown remarkable anti-tumor efficacy against solid tumors, sparking a surge of interest in ADCs with camptothecin derivatives as ADC effector molecules. In this study, we introduced and successfully constructed quaternary ammonium ADCs utilizing camptothecin derivatives **WL-14** and **CPTS-1** for the first time. All four ADCs displayed excellent stability under physiological conditions and in plasma, facilitating their prolonged circulation *in vivo*. Moreover, the four ADCs, employing Val-Cit or Val-Ala dipeptide linkers effectively achieved complete release of the effector molecules via cathepsin B. Although, the *in vitro* antitumor activity of these ADCs was comparatively limited, the development of quaternary ammonium ADCs based on novel camptothecin derivatives as effector molecules is still a viable and promising strategy. Significantly, our study provides valuable insights into the crucial role of linker optimization in ADCs design.

## Introduction

**Fig 1:**
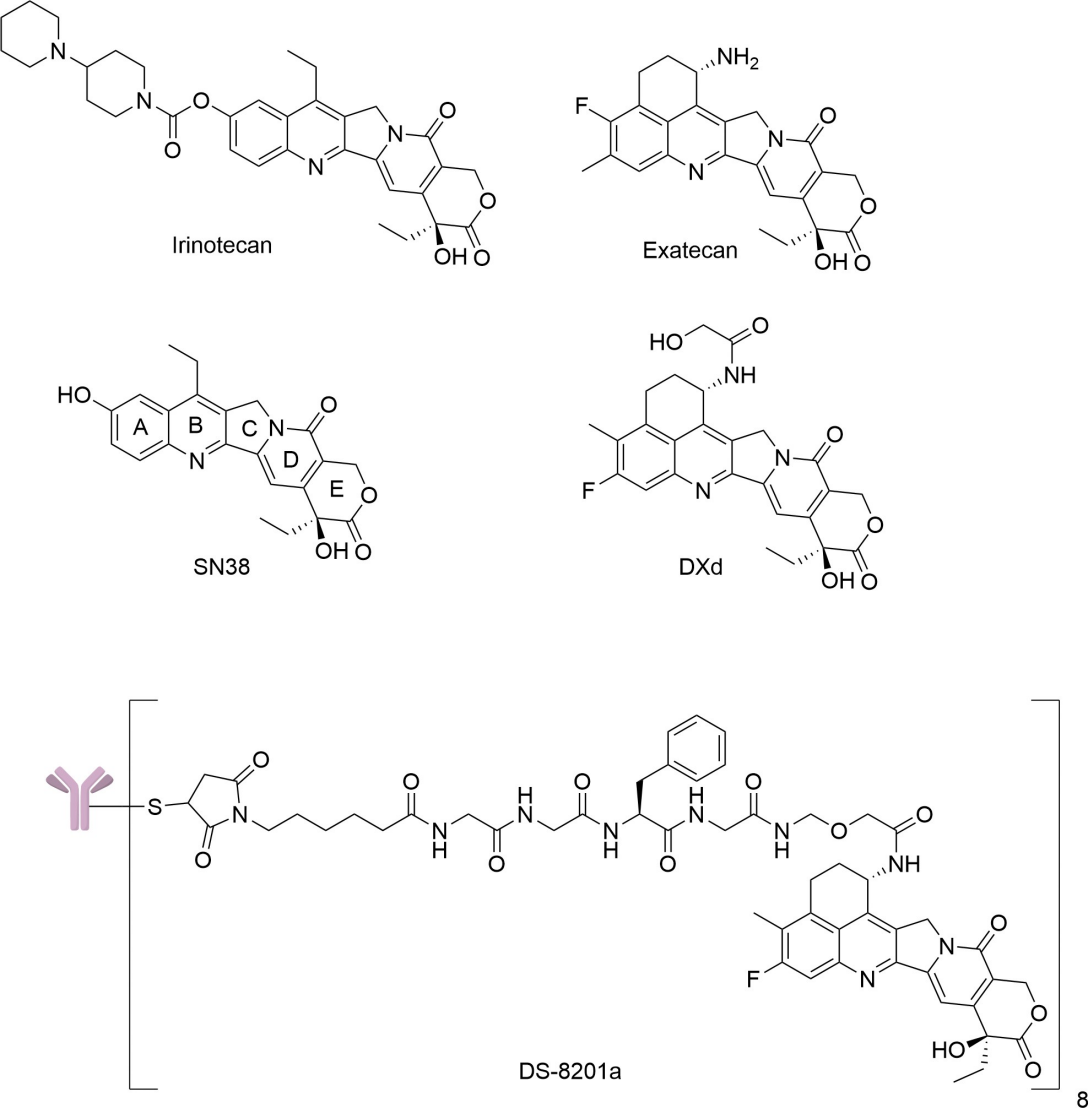
Structures of camptothecin derivatives.

Antibody drug conjugates (ADCs) are currently a cutting-edge targeted drug delivery systems with high anti-tumor potency, low toxicity and prolonged circulation *in vivo*, which have demonstrated remarkable therapeutic effects in the clinic [1–4]. ADCs mainly consist of recombinant monoclonal antibodies covalently bound to cytotoxic effector molecules through various linkers [5]. The development of novel ADCs relies on the continuous improvement of monoclonal antibody and linker technologies, while the effector molecules are the core element of novel ADC development. DS-8201a (Fig 1), an ADC approved for marketing by the FDA in 2019, employed the camptothecin derivative DXd as an effector molecule to conjugate with trastuzumab targeting HER2 via a tetrapeptide linker (GGFG) [6, 7]. The remarkable therapeutic effects of DS-8201a on solid tumors, including HER2-positive breast cancer, gastric cancer, and non-small cell lung cancer, indicate the enormous potential of camptothecin derivatives as ADC effector molecules [4, 8–10], making the development of novel camptothecin derivatives the hottest research direction in effector molecules. However, the gastrointestinal and haematological side effects exhibited by DS-8201a in clinical applications, such as nausea, vomiting, and anaemia, have greatly limited its use, which are mainly attributed to DXd [11]. The major challenge for ADCs based on camptothecin derivatives is to expand the therapeutic window. Strategies to address this issue include selecting more cytotoxic camptothecin derivatives to reduce the minimum effective dose or identifying camptothecin derivatives with a higher *in vivo* safety profile to increase the maximum tolerated dose.

Currently, the main camptothecin derivatives applied as ADC effector molecules are SN38 and DXd (Fig 1), which specifically bind to topoisomerase I, interfere with DNA transcription and replication, and ultimately cause cell death by forming a stable ternary complex with DNA [12–16]. SN38 is the active metabolite of irinotecan and DXd is the active metabolite of DS-8201a, both of which are promising candidates for ADC effector molecules [17–21]. Among these reported ADCs, linkers such as ester bond, carbonate [22], carbamate [23], β-glucuronide [24], AcLys-ValCit-PABC [25], Val-Ala-PABC [26], Ala-Ala-Ala [9], and GGFG [27] are usually used to link to effector molecules with primary, secondary amines or hydroxyl groups, and no relevant ADCs have been reported for highly cytotoxic camptothecin derivatives without connecting sites. Pillow et al. reported a quaternary ammonium ADC based on tubulysin, a linker that ensured stable connection and traceless release of tertiary amines and exhibited excellent antitumor activity *in vitro* and *in vivo* [28]. Liao et al. designed a novel ADC conjugated immunomodulator D18 with anti-PD-L1 antibody via a quaternary ammonium linker [29]. This provides us with a new strategy to prepare ADCs from camptothecin derivatives without linking sites by quaternary ammonium linker, and here we designed and synthesized four quaternary ammonium ADCs with camptothecin derivatives as payloads.

## Results and discussion

### Design and synthesis linker-drug complexes

**Fig 2:**
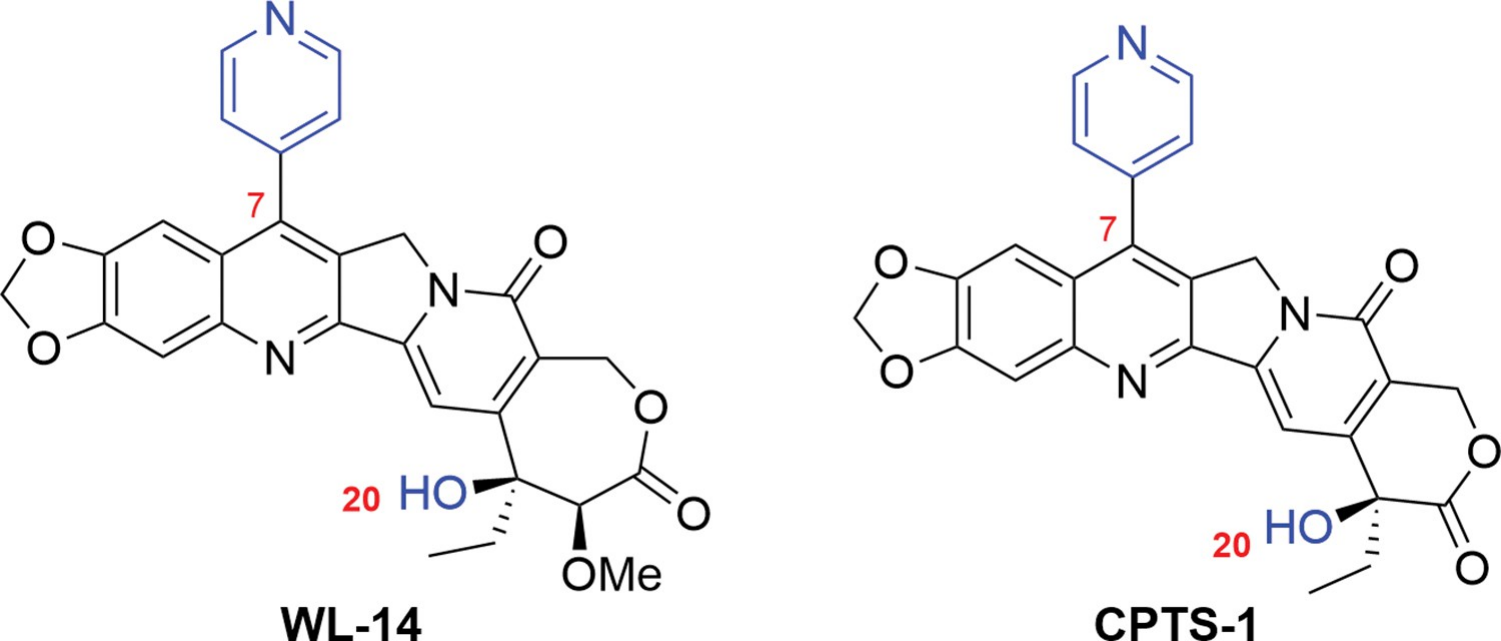
Structures of novel camptothecin derivatives, where the blue groups indicate the available connecting sites.

In the development of camptothecin derivatives, previous studies have demonstrated that the incorporation of aryl heterocycles such as pyridine, methylpyrazole, and indazole at the 7-position of camptothecin could significantly enhance the cytotoxic activity of these derivatives [30]. Building upon this knowledge, we previously introduced a pyridine group at the 7-position of homocamptothecin to synthesize **WL-14**, which showed potent *in vivo* anti-tumor effects and demonstrated good safety profiles in S180 and HT-29 tumor-bearing mice while maintaining high cytotoxic activity, and is an ideal camptothecin derivative for ADC [31]. The traditional camptothecin derivative **CPTS-1** was also prepared by retaining the piperidine ring and pyridine ring structure of **WL-14** and converting the seven-membered ring to the traditional six-membered ring (Fig 2).

Both the 7-position pyridine group and the 20-position hydroxyl group in the structures of CPTS-1 and WL-14 can serve as sites for conjugation (Fig 2). However, due to the high steric hindrance and limited reactivity of the tertiary alcohol at the 20-position, the pyridine group was chosen as the preferred site for conjugation. Additionally, to validate the feasibility of these two novel classes of camptothecin derivatives as ADCs, the researchers adopted a previously reported linking method and dipeptide linkers to minimize the impact of other factors on the potency of the resulting ADCs. Referring to the work by Pillow’s group [32], the quaternary ammonium linker was considered a suitable connecting strategy, and linker-drug complexes suitable for ADCs were synthesized by generating quaternary ammonium salts at the 7-position pyridine in this study.

### Chemistry

**Fig 3:**
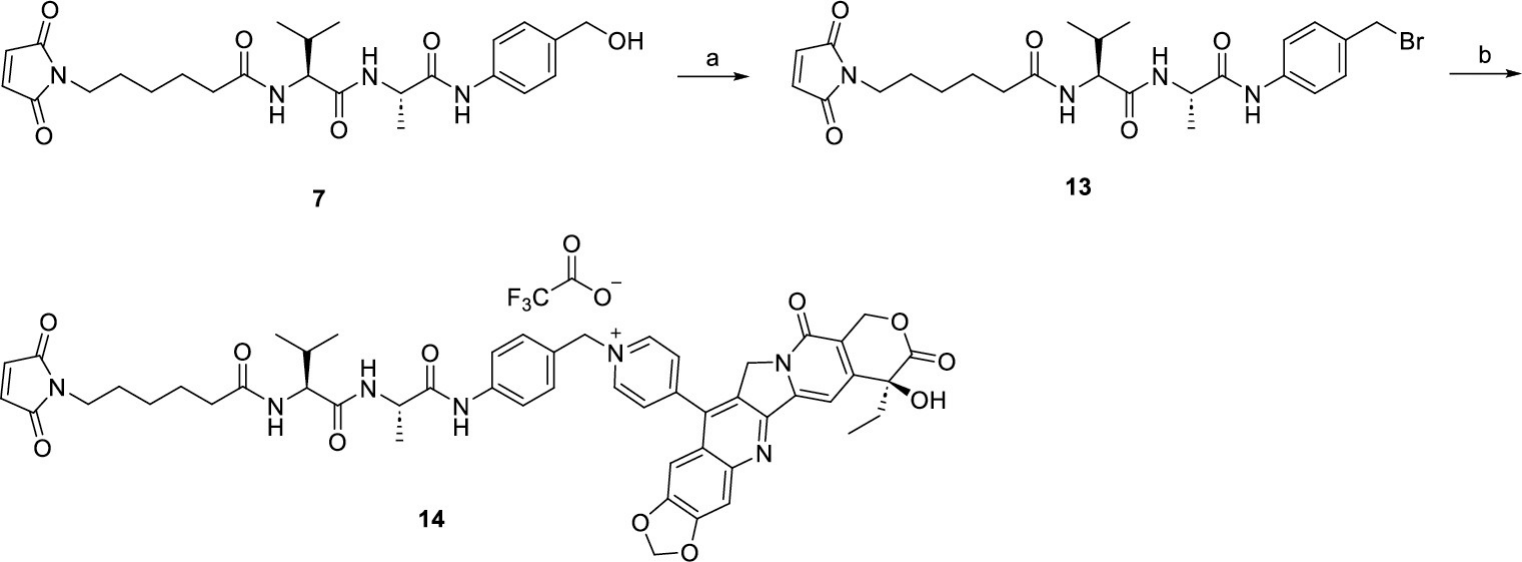
Reagents and conditions: (a) PBr_3_, THF, rt, 4 h; (b) CPTS-1, DMF, rt, 24 h, 45%.

**Fig 4:**
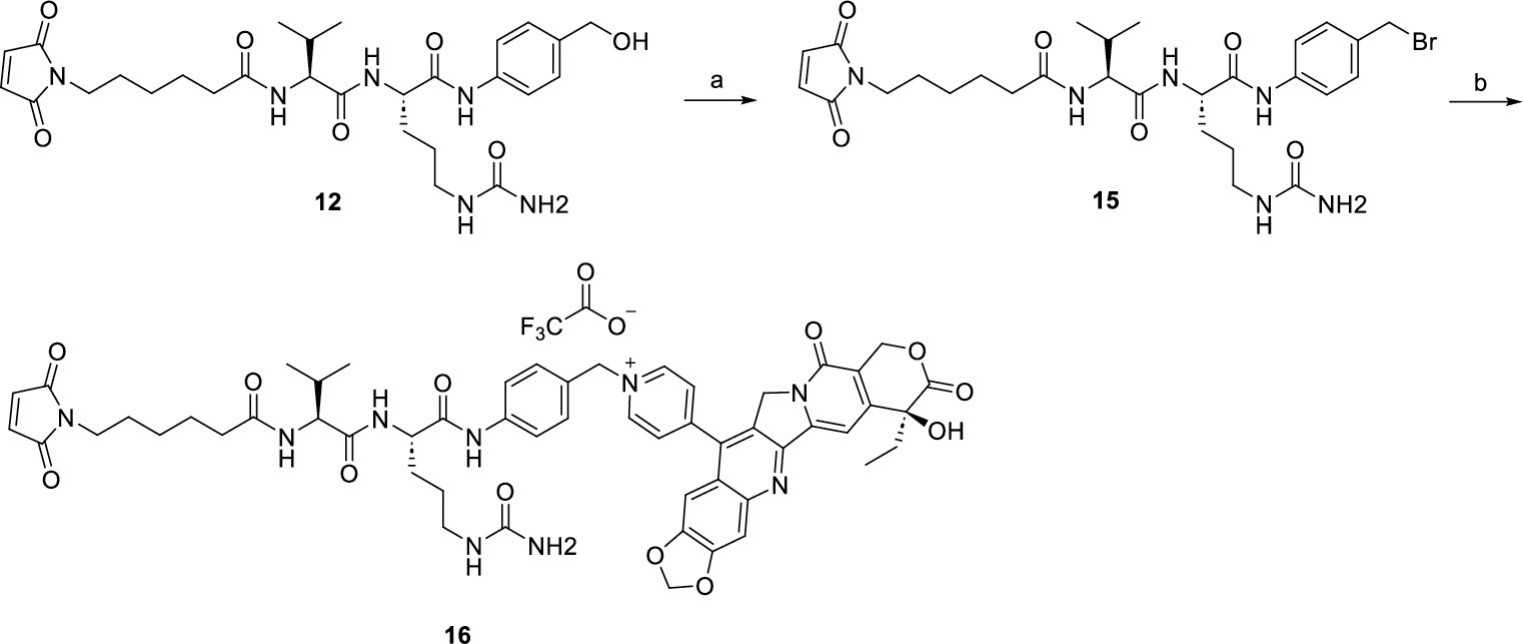
Reagents and conditions: (a) PBr_3_, THF, rt, 4 h; (b) CPTS-1, DMF, rt, 24 h, 40%.

**Fig 5:**
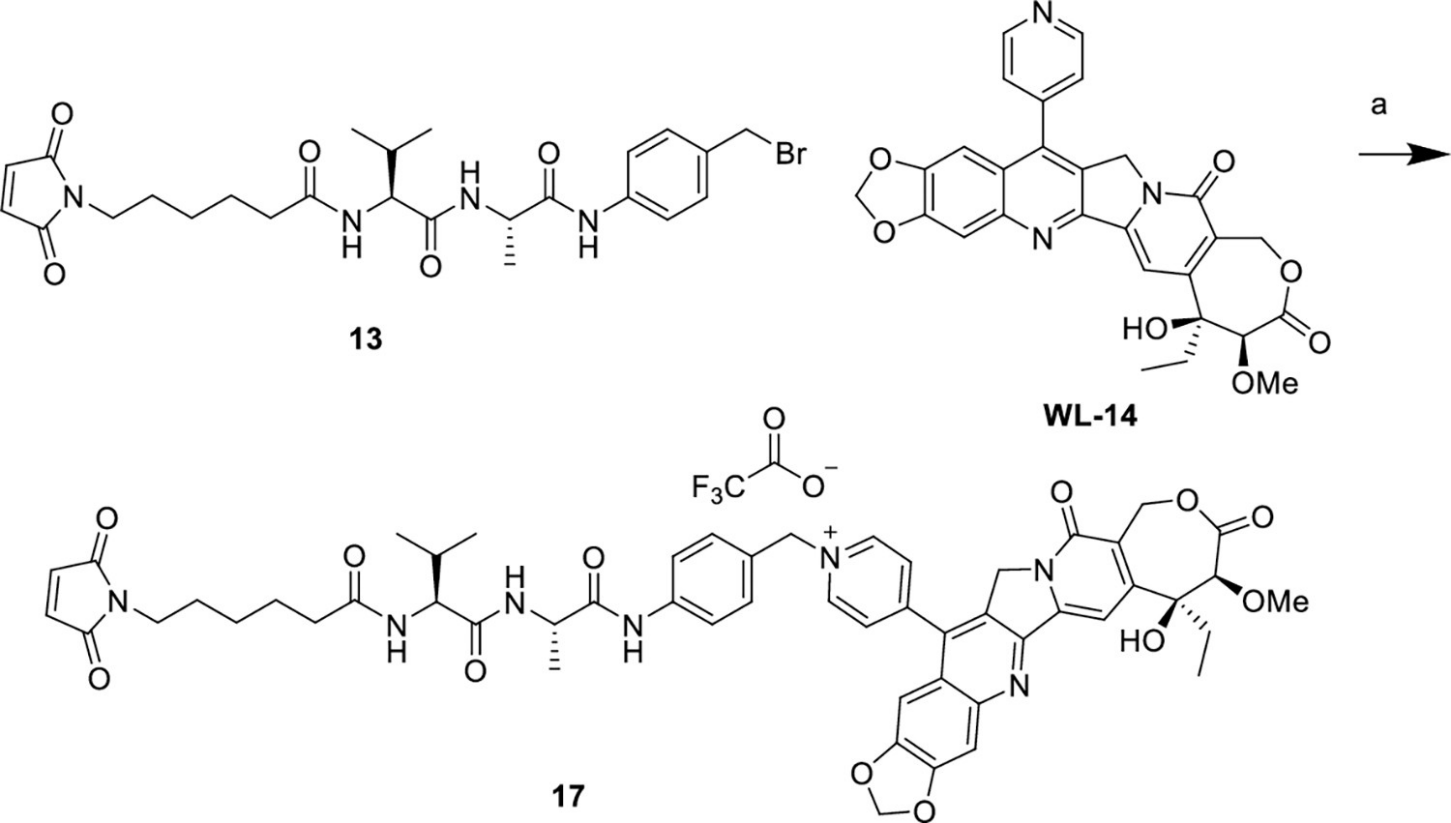
Reagents and conditions: (a) WL-14, DMF, rt, 24 h, 46%.

**Fig 6:**
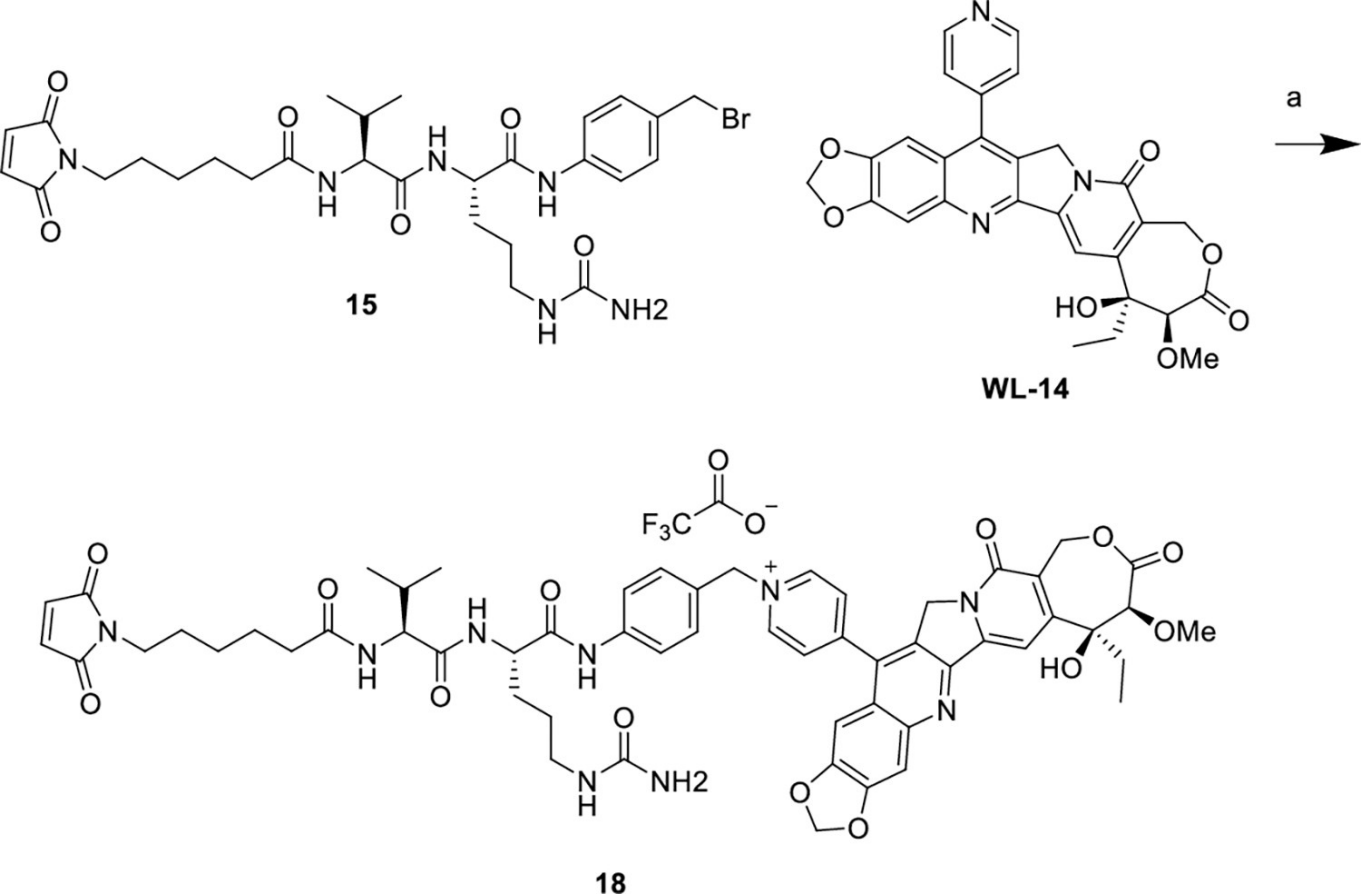
Reagents and conditions: (a) WL-14, DMF, rt, 24 h, 50%.

In S1-S2 Schemes in S1 File and Figs 3–6, we synthesized novel linker-drug complexes based on camptothecin derivative suitable for the construction of ADCs. Firstly, we prepared the dipeptide linkers reported in the literature using commercially available L-alanine and L-citrulline (S1-S2 Schemes in S1 File). It is widely recognized that the p-amino benzyl ether fragment is the most commonly employed spacer group for peptide linkers, as it can undergo 1, 6-elimination under specific conditions, resulting in the release of the effector molecules [33]. Therefore, compounds **1** and **8** were condensed with compound **2**, leading to the formation of compounds **3** and **9**. Subsequently, these compounds were reacted with p-amino benzyl alcohol and the Fmoc group was removed using diethyl amine, yielding compounds **5** and **11**. Finally, compounds **5** and **11** were conjugated with commercially available compound **6**, resulting in the synthesis of the desired dipeptide linkers valine-alanine (Val-Ala) and valine-citrulline (Val-Cit).

**Fig 7:**
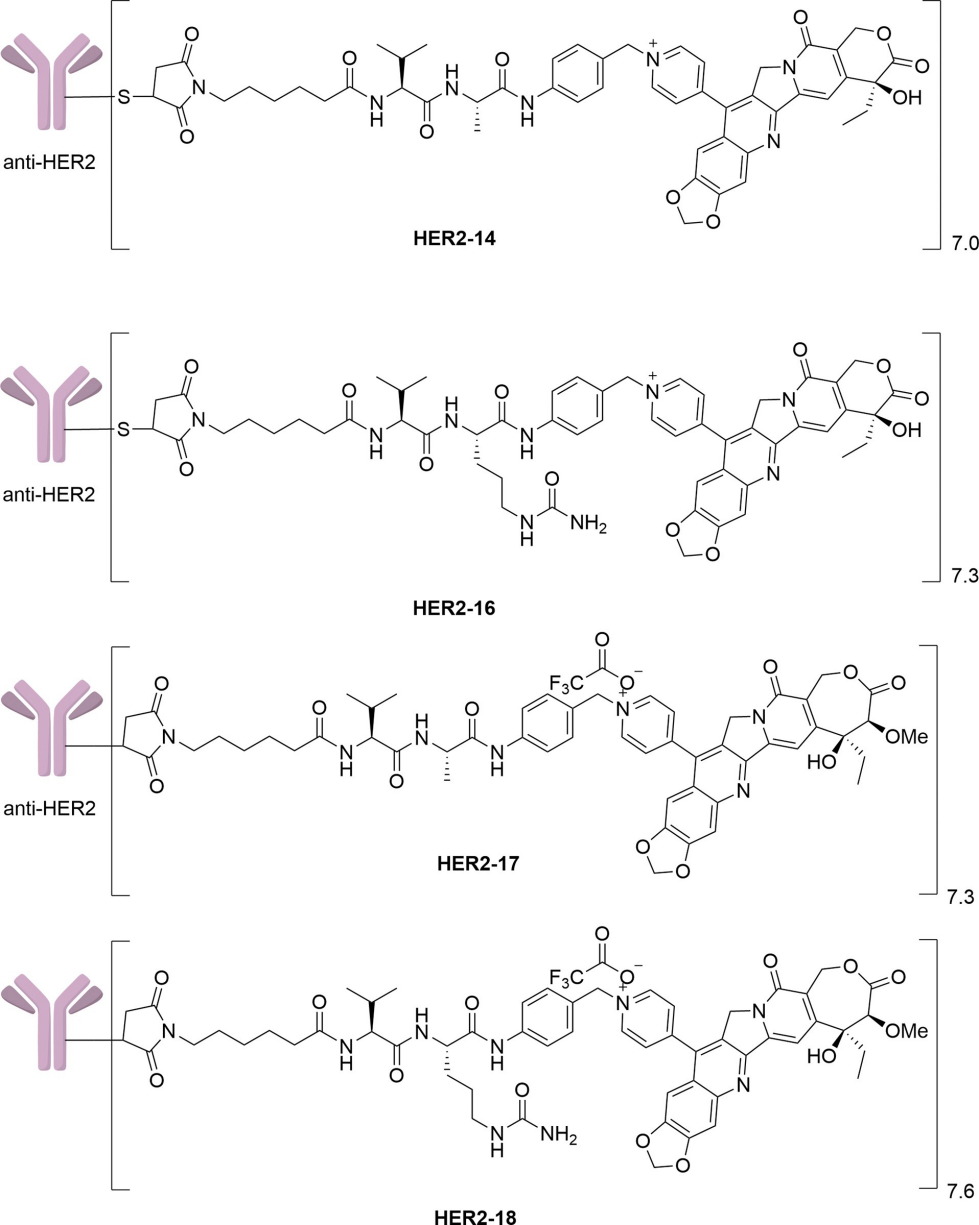
Structures of quaternary ammonium salt-based antibody-drug conjugates based on CPTS-1 and WL-14.

After synthesizing the target dipeptide linkers, we proceeded to brominate compound **7** using phosphorus tribromide, resulting in compound **13**. Compound **13** was then reacted with CPTS-1 in anhydrous DMF at room temperature, and the resulting product was isolated through semi-preparative purification, yielding the desired linker-drug complex **14** (Fig 3). Similarly, for the valine-citrulline dipeptide linker, compound **15** underwent the same reaction with **CPTS-1** to form a quaternary ammonium salt, leading to the formation of the target product **16** (Fig 4). Furthermore, we have depicted the structures of the ADCs **HER2-14** and **HER2-16**, which were prepared by coupling CPTS-1 as the effector molecule to Trastuzumab. The conjugates **HER2-14** and **HER2-16** exhibited drug-to-antibody ratios (DAR) of 7.0 and 7.3 (Fig 7), respectively. Taking reference from the synthesis of compound **14** and compound **16**, we successfully obtained dipeptide linker-drug complexes **17** and **18** with **WL-14** as the effector molecule in Fig 5 and 6, respectively. These complexes were subsequently conjugated with Trastuzumab, resulting in the formation of ADCs HER2-17 and HER2-18 with DAR values of 7.3 and 7.6 (Fig 7), respectively.

### *In vitro* anti-proliferative inhibition assay

We have previously demonstrated that **CPTS-1** and **WL-14** possessed significant cytotoxic activity, making them suitable candidates for the development of ADCs targeting the HER2 [31, 34]. **CPTS-1** and **WL-14** were then tested for *in vitro* cytotoxic activity in HER2-positive cancer cells (human gastric cancer NCI-N87 and human breast cancer SK-BR-3), as well as HER2-negative cells (human breast cancer cells MDA-MB-468). Exatecan and DXd were selected as positive controls. The results revealed that both **CPTS-1** and **WL-14** maintained potent cytotoxic activity in both HER2-positive and HER2-negative tumor cells, indicating their potential as ADC payloads (Table 1).

**Table 1:** Anti-proliferative activity of CPTS-1 and WL-14 against various tumor cells with different expression of HER2.

	IC_50_ (nM, Mean±SD, n = 3)		
**Compounds**	**NCI-N87**	**SK-BR-3**	**MDA-MB-468**
**CPTS-1**	<0.05	<0.013	<0.05
**WL-14**	<0.013	<0.013	<0.013
**Exatecan**	2.70 ± 0.97	1.97 ± 0.36	1.22 ± 0.89
**DXd**	9.11 ± 2.33	3.77 ± 0.17	3.11 ± 0.20

### The stability analysis of linker-drug complexes

After considering **CPTS-1** and **WL-14** as potential effector molecules for ADCs, we conducted stability analyses on their linker-drug complexes connected by Val-Ala and Val-Cit to preliminarily evaluate their stability in the body circulation. Excitingly, our findings demonstrated that all complexes maintained their stability under PBS 7.4 conditions for a duration of 7 days. The maximum release observed was 1.72% for **CPTS-1** and a mere 0.91% for **WL-14** (Table 2). These results showed the exceptional stability of these complexes, highlighting their potential for use in ADCs.

**Table 2:** The stability of linker-drug complexes 14 and 16 in PBS.

Compounds	CPTS-1 (%)			
	Day 1	Day 3	Day 5	Day 7
**14**	/^a^	/^a^	0.47± 0.01	0.95 ± 0.01
**16**	/^a^	1.03 ± 0.04	1.35 ± 0.03	1.72 ± 0.09
**17**	/^b^	/^b^	/^b^	0.72 ± 0.01
**18**	/^b^	/^b^	0.43 ± 0.01	0.91 ± 0.02

^a^ "/" represents that no **CPTS-1** release was detected.

^b^ "/" represents that no **WL-14** release was detected.

### The enzyme release experiments of ADCs

**Fig 8:**
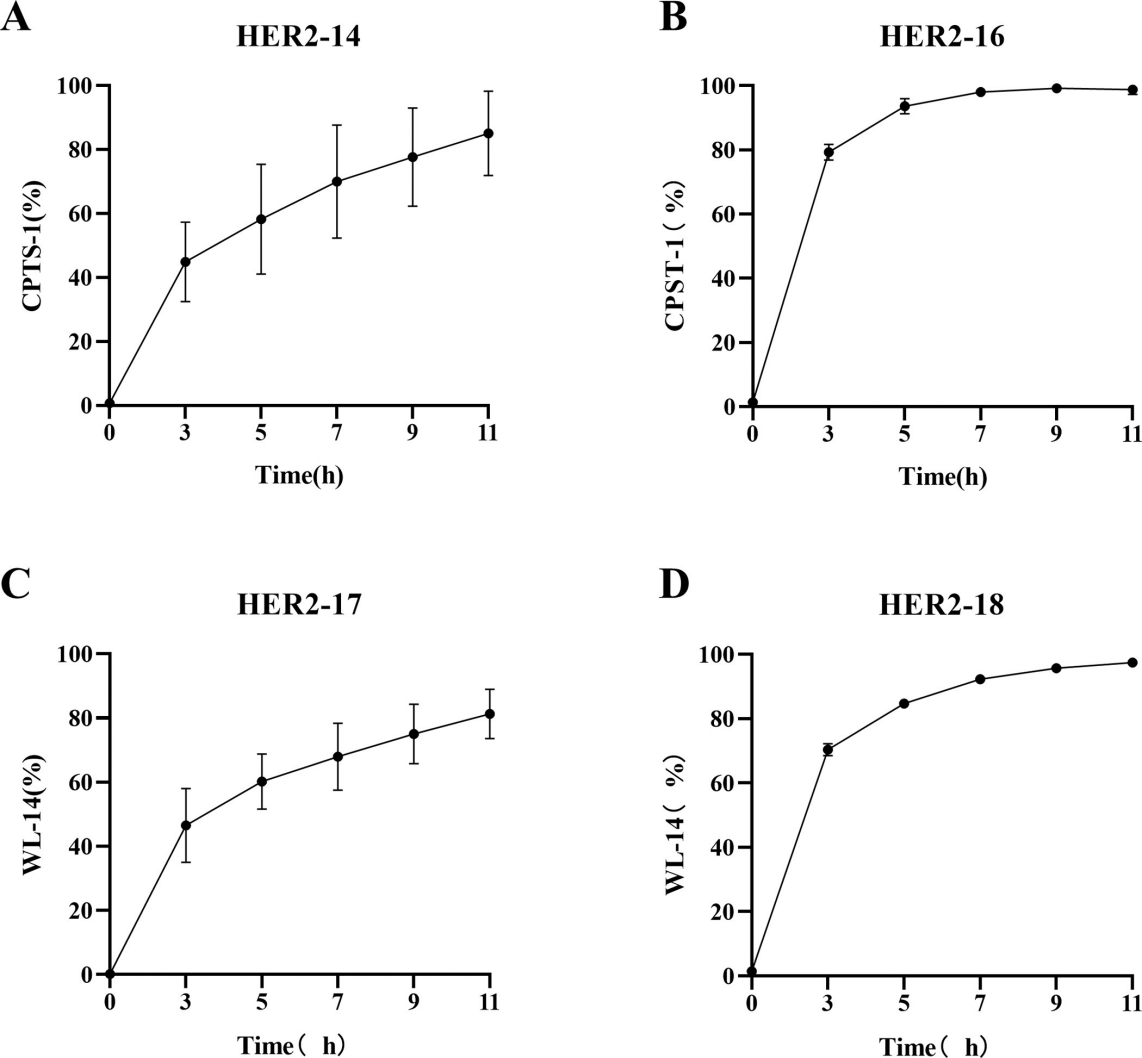
The release of CPTS-1 and WL-14 from ADCs via cathepsin B.

To further validate the viability of releasing peptide linkers with quaternary ammonium structures, we conducted *in vitro* enzymatic release experiments using cathepsin B on **HER2-14**, **HER2-16** ~ **HER2-18**, as depicted in Fig 8. Remarkably, in the presence of cathepsin B, the ADCs comprising the Val-Ala dipeptide linker achieved complete release of the effector molecule within 5 hours. Similarly, the ADCs featuring the Val-Cit dipeptide linker achieved complete release within 4 hours. Mass spectra of four **ADCs** before and after cleavage by cathepsin B are also supplied in the S1 File (S9-S12 Figs), further demonstrating that ADCs can be cleaved by cathepsin B. In addition, we also performed enzymatic release experiments on linker-drug complexes and observed a similar phenomenon. Free camptothecin was released more slowly by linker-drug complexes connected by Val-Ala dipeptide linkers (S13 Fig in S1 File). These results demonstrated the efficient and rapid release capabilities of our peptide quaternary ammonium linkers, further supporting their potential as valuable components in drug delivery systems.

### The biological analysis of ADCs

According to the above experimental data presented, further biological evaluation of the ADCs was carried out. The weighted average DAR and SEC values of the ADCs were first determined in **Table 3** provided the weighted average DAR and size-exclusion chromatography (SEC) values of the ADCs. The DAR values of the ADCs ranged from 7.0 to 7.6, all exhibiting an average aggregation rate of less than 8%. These results aligned with the established criteria for ADCs, as described in S1 File (S1-S8 Figs, S1-S4 Tables).

**Table 3:** The weighted average DAR and SEC values of four ADCs.

ADCs	DAR (drug: antibody)	SEC (%)
**HER2-14**	7.0	96.46%
**HER2-16**	7.3	92.94%
**HER2-17**	7.3	95.42%
**HER2-18**	7.6	96.26%

In previous studies, it was observed that all linker-drug complexes released minimal amounts of **CPTS-1** or **WL-14** during stability experiments conducted over a period of 7 days in PBS (pH = 7.0). To confirm the high stability of these compounds in plasma, the corresponding ADCs were subjected to stability testing in mouse plasma. The results in Table 4 demonstrated that neither of the ADCs released more than 1% of **CPTS-1** or **WL-14**.

**Table 4:** The stability of ADCs HER2-14 and HER2-16 in mouse plasma.

Compounds	CPTS-1 (%)			
	Day 1	Day 3	Day 5	Day 7
**HER2-14**	/^a^	0.19 ± 0.01	0.31 ± 0.04	0.54 ± 0.01
**HER2-16**	0.11 ± 0.01	0.32 ± 0.05	0.51 ± 0.06	0.74 ± 0.01
**HER2-17**	0.13 ± 0.01	0.21 ± 0.02	0.35 ± 0.05	0.48 ± 0.01
**HER2-18**	0.10 ± 0.02	0.22 ± 0.03	0.50 ± 0.10	0.72 ± 0.08

^a^ "/" represents that no **CPTS-1** release was detected.

### *In vitro* anti-proliferative inhibition assay of ADCs

Subsequently, we tested the anti-proliferative activity of both ADCs *in vitro* in HER2-positive NCI-N87 cells. All ADCs exhibited cytotoxic activities at several dozens of nonomolar level after co-incubation with cells for 120 h (Table 5). Although we successfully prepared novel quaternary ammonium ADCs **HER2-14** and **HER2-16** based on **CPTS-1** and **HER2-17** and **HER2-18** based on **WL-14**, the *in vitro* cytotoxic activity was relatively limited. Linker-drug complexes have been shown to be effective in releasing the effector molecule in the presence of cathepsin B. We therefore hypothesized that the limited cytotoxic activity exhibited by ADCs was due to the limited intracellular release of the 1,6-elimination process of the quaternary ammonium spacer, resulting in inability to release **CPTS-1** or **WL-14** promptly and thus exhibiting weaker cellular activity. Another reason might be that novel camptothecin derivatives containing pyridine moiety were not suitable for release in intracellular lysosomes, and the low pH might have an impact on properties such as the membrane permeability of **CPTS-1** or **WL-14**, resulting in its inability to kill tumor cells effectively.

**Table 5:** Anti-proliferative activity of HER2-14 against NCI-N87 cells.

Compounds	EC50^a^ (ng/ml, Mean±SD, n = 3)
	NCI-N87, 120 h
**HER2-14**	145.43 ± 27.69
**HER2-16**	166.41 ± 39.34
**HER2-17**	142.21 ± 26.91
**HER2-18**	67.94 ± 13.53

^a^ EC_50_ values represent the mean of at least three independent experiments.

## Conclusion

In this study, we primarily validated the rationality of novel camptothecin derivatives as effector molecules for the development of corresponding ADCs. We successfully synthesized four quaternary ammonium ADCs by linking camptothecin derivatives **CPTS-1** and **WL-14** to trastuzumab via Val-Ala and Val-Cit dipeptide quaternary ammonium linkers, which can be cleaved by cathepsin B. In comparison to the positive control DXd, both **CPTS-1** and **WL-14** demonstrated superior cytotoxic activity against HER2-positive cancer cells. While these compounds exhibited enhanced *in vitro* anti-proliferation activity compared to Exatecan and DXd and were considered as potential candidates for effector molecules based on their cellular activity, the corresponding ADCs displayed limited *in vitro* antitumor activity. This observation may be attributed to the limited intracellular release of quaternary ammonium linkers and the adverse impact of pyridine group-containing camptothecin derivatives on membrane permeability under conditions of low intracellular pH. In conclusion, camptothecin derivatives **CPTS-1** and **WL-14** held promise for application in quaternary ammonium ADC development. Significantly, our study provided valuable insights into the crucial role of linker optimization in ADCs design.

## Materials and methods

### General methods

The ^1^H and ^13^C NMR spectra of all compounds in this paper were obtained by Bruker Ascend^TM^-400 MHz Fourier transform NMR tester. The solvents used were CDCl_3_-*d*, DMSO-*d*_6_, Methanol-*d*_4_ and Acetic Acid-*d*_4_, the chemical shifts (δ) are in ppm and the coupling constants (*J*) are in Hz. The ionization source for low-resolution mass spectrometry was electrospray ionization (ESI), measured by a Waters ACQUITY UPLC SQ MS. The ionization source for high-resolution mass spectrometry was electrospray ionization (ESI), measured by an IonSpec 4.7 Tesla FTMA mass spectrometer. The HPLC (High Performance Liquid Chromatography) detection instruments involved in this paper include Agilent 1200 and DGU-20A5R High Performance Liquid Chromatography. Melting points were determined using the SGW X-4 micro melting point instrument.

#### General procedure for the synthesis of compounds 7 and 12

*(((9H-fluoren-9-yl)methoxy)carbonyl)-L-valyl-L-alanine* **3**. Compound **1** (3.0 g, 34.4 mmol) was added to 150 mL of mixed solvent (H_2_O: THF = 1:1) and stirred for 5 min. Then NaHCO_3_ (3.6 g, 34.4 mmol) was added and the system became clear and transparent. Compound **2** (15.0 g, 34.4 mmol) was dissolved in DME (120 mL) and then slowly added to the reaction system, and the reaction was carried out overnight at room temperature. After the reaction, 1 M hydrochloric acid was added to adjust the pH to 2. A large amount of solid was precipitated and filtered. The solid was dissolved with a large amount of EA and isopropanol (10:1) mixture solvent and the insoluble material was filtered off. The filtrate was washed with water several times and evaporated to obtain a waxy solid, which was repeatedly beaten with a mixture of DCM and EA to obtain a white solid. ^1^H NMR (400 MHz, DMSO-*d*_6_) δ 12.52 (s, 1H), 8.24 (d, J = 6.8 Hz, 1H), 7.90 (d, J = 7.5 Hz, 2H), 7.76 (t, J = 6.5 Hz, 2H), 7.42 (t, J = 7.0 Hz, 3H), 7.33 (t, J = 7.2 Hz, 2H), 4.24 (qd, J = 11.8, 3.5 Hz, 4H), 3.90 (t, J = 8.1 Hz, 1H), 1.98 (h, J = 6.8 Hz, 1H), 1.28 (d, J = 7.3 Hz, 3H), 0.89 (dd, J = 14.1, 6.7 Hz, 6H). Yield: 7.0 g, 50%. Melting point: 160–162°C. MS (ESI) m/z = 411.5 (M + H^+^).

*(9H-fluoren-9-yl)methyl ((S)-1-(((S)-1-((4-(hydroxymethyl)phenyl)amino)-1-oxopropan-2-yl)amino)-3-methyl-1-oxobutan-2-yl)carbamate* **4**. Compound **3** (5.0 g, 12.2 mmol) and p-aminobenzyl alcohol (3.0 g, 24.4 mmol) were dissolved in 210 mL of a mixture solvent (DCM: MeOH = 2:1) and EEDQ (6.0 g, 24.4 mmol) was added and reacted overnight at room temperature. After the reaction, the crude product was filtered directly and purified with anhydrous ether to give a white solid. ^1^H NMR (400 MHz, DMSO-*d*_6_) δ 9.93 (s, 1H), 8.17 (d, J = 7.0 Hz, 1H), 7.89 (d, J = 7.5 Hz, 2H), 7.75 (t, J = 7.2 Hz, 2H), 7.54 (d, J = 8.1 Hz, 2H), 7.43 (q, J = 8.4, 7.3 Hz, 3H), 7.33 (t, J = 7.3 Hz, 2H), 7.24 (d, J = 8.2 Hz, 2H), 5.10 (d, J = 5.0 Hz, 1H), 4.43 (d, J = 3.8 Hz, 3H), 4.30 (d, J = 10.2 Hz, 1H), 4.23 (s, 2H), 3.92 (t, J = 7.9 Hz, 1H), 2.05–1.95 (m, 1H), 1.31 (d, J = 7.0 Hz, 3H), 0.88 (dd, J = 12.2, 6.7 Hz, 6H). Yield: 5.0 g, 80%. Melting point: 131–133°C. MS (ESI) m/z = 516.5 (M + H^+^).

*(S)-2-amino-N-((S)-1-((4-(hydroxymethyl)phenyl)amino)-1-oxopropan-2-yl)-3-methylbutanamide* **5**. Compound **4** (1.0 g, 1.94 mmol) was dissolved in 10 mL of anhydrous DMF, 2 mL of diethylamine was added and the reaction was stirred at room temperature for 3 h. After the reaction, the DMF and diethylamine were removed under reduced pressure to give a viscous liquid. The mixture was stirred overnight and filtered to give a white solid. ^1^H NMR (400 MHz, DMSO-*d*_6_) δ 9.99 (s, 1H), 8.17 (s, 1H), 7.54 (d, J = 8.3 Hz, 2H), 7.25 (d, J = 8.3 Hz, 2H), 4.47 (d, J = 5.8 Hz, 1H), 4.44 (s, 2H), 3.18 (s, 1H), 3.02 (d, J = 4.8 Hz, 1H), 2.51 (s, 1H), 1.97–1.89 (m, 1H), 1.31 (d, J = 7.0 Hz, 3H), 0.89 (d, J = 6.8 Hz, 3H), 0.79 (d, J = 6.8 Hz, 3H). Yield: 400 mg, 70%. Melting point: 133–135°C. MS (ESI) m/z = 294.3 (M + H^+^).

*6-(2*,*5-dioxo-2*,*5-dihydro-1H-pyrrol-1-yl)-N-((S)-1-(((S)-1-((4-(hydroxymethyl)phenyl)amino)-1-oxopropan-2-yl)amino)-3-methyl-1-oxobutan-2-yl)hexanamide* **7**. Compound **5** (400 mg, 1.36 mmol) and compound **6** (460 mg, 1.50 mmol) were dissolved in 15 mL of anhydrous THF and reacted for 12 h at room temperature. After the reaction, the solvent was removed and the crude product was separated by column chromatography (DCM: MeOH = 10:1) to give a white solid. Yield: 530 mg, 80%. ^1^H NMR (400 MHz, DMSO-*d*_6_) δ 9.86 (s, 1H), 8.15 (d, J = 6.7 Hz, 1H), 7.83 (d, J = 8.5 Hz, 1H), 7.54 (d, J = 7.9 Hz, 2H), 7.23 (d, J = 7.9 Hz, 2H), 7.01 (s, 2H), 5.10 (t, J = 5.5 Hz, 1H), 4.42 (d, J = 5.4 Hz, 2H), 4.41–4.36 (m, 1H), 4.17 (t, J = 7.5 Hz, 1H), 3.37 (d, J = 6.9 Hz, 2H), 2.14 (tt, J = 13.8, 6.9 Hz, 2H), 2.00–1.91 (m, 1H), 1.52–1.43 (m, 4H), 1.30 (d, J = 6.8 Hz, 3H), 1.19 (q, J = 7.4 Hz, 2H), 0.86 (d, J = 6.4 Hz, 3H), 0.82 (d, J = 6.4 Hz, 3H). MS (ESI) m/z = 487.4 (M + H^+^).

*(S)-2-((S)-2-((((9H-fluoren-9-yl)methoxy)carbonyl)amino)-3-methylbutanamido)-5-ureidopentanoic acid* **9**. According to the synthesis of compound **3**, white solid.^1^ HNMR (400 MHz, DMSO-*d*_6_): δ 12.53 (s, 1H), 8.18 (d, J = 7.4 Hz, 1H), 7.89 (d, J = 7.4Hz, 2H), 7.75 (t, J = 6.4 Hz, 2H), 7.42 (m, 3H), 7.33 (t, J = 7.4 Hz, 2H), 5.94 (s, 1H), 5.38(s, 2H), 4.35–4.06 (m, 4H), 3.92 (dd, J = 7.4 Hz, 8.8 Hz, 1H), 2.94 (q, J = 6.2 Hz, 2H),1.98 (m, 1H), 1.71–1.38 (m, 4H), 0.87 (m, 6H). Yield: 42%. Melting point: 170–172°C. MS (ESI) m/z = 497.6 (M + H^+^).

*(9H-fluoren-9-yl)methyl ((S)-1-(((S)-1-((4-(hydroxymethyl)phenyl)amino)-1-oxo-5-ureidopentan-2-yl)amino)-3-methyl-1-oxobutan-2-yl)carbamate* **10**. According to the synthesis of compound **4**, white solid. ^1^HNMR (DMSO-*d*_6_): δ 9.98 (s, 1H), 8.11 (d, J = 7.4 Hz, 1H), 7.87 (d, J = 7.4 Hz, 2H), 7.77 (m, 2H), 7.52 (m, 2H), 7.39 (m, 3H), 7.30(m, 2H), 7.21 (m, 2H), 5.97 (m, 1H), 5.41 (s, 2H), 5.10 (m, 1H), 4.42 (m, 3H), 4.22 (m, 3H), 3.90 (m, 1H), 2.93 (m, 2H), 1.98 (m, 1H), 1.50 (m, 2H), 1.30 (m, 2H), 0.84 (m, 6H). MS (ESI) m/z = 602.6 (M + H^+^). Yield: 83%. Melting point: 140–142°C.

*(S)-2-((S)-2-amino-3-methylbutanamido)-N-(4-(hydroxymethyl)phenyl)-5-ureidopentanamide* **11**. According to the synthesis of compound **5**, white solid. ^1^HNMR (400 MHz, DMSO-*d*_6_): δ 10.03 (brs, 1H), 8.12 (brs, 1H), 7.52 (d, J = 8.5 Hz 2H), 7.23 (d, J = 8.2 Hz, 2H), 5.98 (m, 1H), 5.40 (s, 2H), 5.09 (brs, 1H), 4.46 (s, 2H), 3.05 (d, J = 4.7 Hz, 1H), 2.89–3.01 (m, 2H),1.94–1.92 (m, 1H), 1.52–1.68 (m, 2H), 1.38–1.42 (m, 2H), 0.85–0.82 (m, 6H). MS (ESI) m/z = 380.5 (M + H^+^). Yield: 80%. Melting point: 143–145°C.

*6-(2*,*5-dioxo-2*,*5-dihydro-1H-pyrrol-1-yl)-N-((S)-1-(((S)-1-((4-(hydroxymethyl)phenyl)amino)-1-oxo-5-ureidopentan-2-yl)amino)-3-methyl-1-oxobutan-2-yl)hexanamide* **12**. According to the synthesis of compound **6**, white solid. ^1^H NMR (400 MHz, DMSO-*d*_6_) δ 9.93 (s, 1H), 8.05 (d, J = 6.9 Hz, 1H), 7.81 (d, J = 8.0 Hz, 1H), 7.55 (d, J = 7.7 Hz, 2H), 7.22 (d, J = 7.8 Hz, 2H), 6.99 (s, 2H), 6.05 (s, 1H), 5.40 (s, 2H), 5.07 (s, 1H), 4.42 (s, 3H), 4.18 (t, J = 7.2 Hz, 1H), 3.41–3.35 (m, 2H), 2.98 (dd, J = 13.6, 6.2 Hz, 2H), 2.15 (dt, J = 13.1, 7.0 Hz, 2H), 1.98 (dd, J = 12.7, 6.4 Hz, 1H), 1.71 (s, 1H), 1.61 (s, 1H), 1.52–1.44 (m, 4H), 1.43–1.32 (m, 2H), 1.23–1.15 (m, 2H), 0.88–0.80 (m, 6H). MS (ESI) m/z = 573.4 (M + H^+^). Yield: 80%.

#### General procedure for the synthesis of compounds 14 and 16

*N-((S)-1-(((S)-1-((4-(bromomethyl)phenyl)amino)-1-oxopropan-2-yl)amino)-3-methyl-1-oxobutan-2-yl)-6-(2*,*5-dioxo-2*,*5-dihydro-1H-pyrrol-1-yl)hexanamide* **13**. Compound **7** (250 mg, 0.51 mmol) was dispersed in 10 mL of anhydrous THF and phosphorus tribromide (60 μL, 0.61 mmol) was added dropwise to the system in an ice-water bath and the reaction was kept at 0°C for 3 h after the dropwise addition. After the reaction was completed, the system was poured into ice water and a solid was precipitated. The crude product was obtained by filtration without further purified.

*1-(4-((S)-2-((S)-2-(6-(2,5-dioxo-2,5-dihydro-1H-pyrrol-1-yl)hexanamido)-3-methylbutanamido)propanamido)benzyl)-4-((S)-7-ethyl-7-hydroxy-8,11-dioxo-7,8,11,13-tetrahydro-10H-[1,3]dioxolo[4,5-g]pyrano[3’,4’:6,7]indolizino[1,2-b]quinolin-14-yl)pyridin-1-ium* **14**. Compound **7** (160 mg, 0.29 mmol) and **CPTS-1** (55 mg, 0.12 mmol) were dispersed in 2 mL of anhydrous DMF and the reaction was carried out at 45°C for 24 h. After the reaction, the DMF was removed under reduced pressure to obtain the crude product, which was separated by semi-preparative HPLC (ACN: H_2_O = 3:4) to give a yellow solid. ^1^H NMR (400 MHz, Methanol-*d*_4_) δ 9.35 (d, J = 17.9 Hz, 2H), 8.53 (s, 1H), 8.41–8.33 (m, 1H), 7.81 (d, J = 8.5 Hz, 2H), 7.62 (d, J = 8.5 Hz, 2H), 7.44 (s, 1H), 7.40 (s, 1H), 6.95 (s, 1H), 6.75 (s, 2H), 6.19 (s, 2H), 5.97 (s, 2H), 5.50–5.43 (m, 1H), 5.29 (d, J = 16.2 Hz, 1H), 4.96 (s, 2H), 4.45 (q, J = 7.1 Hz, 1H), 4.11 (d, J = 7.1 Hz, 1H), 3.44 (t, J = 7.0 Hz, 2H), 2.28 (t, J = 7.3 Hz, 2H), 2.08 (q, J = 6.9 Hz, 1H), 1.89 (q, J = 7.3 Hz, 2H), 1.58 (dp, J = 31.1, 7.3 Hz, 4H), 1.45 (d, J = 7.1 Hz, 3H), 1.34–1.23 (m, 3H), 0.96 (dt, J = 16.9, 6.9 Hz, 9H). ^13^C NMR (100 MHz, Methanol-*d*_4_) δ 176.56, 174.63, 173.89, 173.38, 172.60, 158.80, 154.03, 153.81, 152.45, 152.11, 150.70, 149.25, 147.07, 146.90, 141.64, 137.43, 135.33, 131.40, 130.42, 129.50, 127.74, 124.04, 121.90, 120.15, 106.39, 104.83, 100.90, 99.01, 74.23, 66.60, 65.61, 60.80, 51.24, 50.90, 49.74, 49.53, 49.31, 49.10, 48.88, 38.44, 36.39, 32.07, 31.61, 29.42, 27.49, 26.36, 19.73, 18.93, 17.85, 8.16. Yield: 55 mg, 45%. HRMS (ESI) m/z calcd for C_56_H_62_N_9_O_13_^+^ [M^+^]: 938.3725; found 938.3691.

*N-((S)-1-(((S)-1-((4-(bromomethyl)phenyl)amino)-1-oxo-5-ureidopentan-2-yl)amino)-3-methyl-1-oxobutan-2-yl)-6-(2*,*5-dioxo-2*,*5-dihydro-1H-pyrrol-1-yl)hexanamide* **15**. According to the synthesis of compound **13**.

*1-(4-((S)-2-((S)-2-(6-(2,5-dioxo-2,5-dihydro-1H-pyrrol-1-yl)hexanamido)-3-methylbutanamido)-5-ureidopentanamido)benzyl)-4-((S)-7-ethyl-7-hydroxy-8,11-dioxo-7,8,11,13-tetrahydro-10H-[1,3]dioxolo[4,5-g]pyrano[3’,4’:6,7]indolizino[1,2-b]quinolin-14-yl)pyridin-1-ium* **16**. According to the synthesis of compound **14**, yellow solid. ^1^H NMR (400 MHz, Methanol-*d*_4_) δ 10.01 (s, 1H), 9.35 (d, J = 16.8 Hz, 2H), 8.52 (s, 1H), 8.38 (s, 1H), 8.32 (d, J = 7.3 Hz, 1H), 8.04 (d, J = 7.4 Hz, 1H), 7.80 (d, J = 7.8 Hz, 2H), 7.62 (d, J = 8.4 Hz, 2H), 7.45 (s, 1H), 7.41 (s, 1H), 6.94 (s, 1H), 6.77 (s, 2H), 6.19 (s, 2H), 5.97 (s, 2H), 5.47 (d, J = 19.1 Hz, 1H), 5.29 (d, J = 16.3 Hz, 1H), 4.97 (s, 2H), 4.50 (q, J = 7.4, 6.5 Hz, 1H), 4.17–4.11 (m, 1H), 3.45 (t, J = 7.1 Hz, 2H), 3.24–3.06 (m, 2H), 2.28 (t, J = 7.3 Hz, 2H), 2.07 (dq, J = 13.3, 6.6 Hz, 1H), 1.89 (q, J = 7.0, 6.6 Hz, 3H), 1.81–1.70 (m, 1H), 1.58 (dp, J = 29.0, 7.3 Hz, 6H), 1.34–1.23 (m, 5H), 1.00–0.86 (m, 10H). ^13^C NMR (100 MHz, Methanol-*d*_4_) δ 176.48, 174.65, 174.17, 172.66, 172.60, 162.35, 158.78, 154.03, 153.80, 152.44, 152.10, 150.69, 149.25, 147.06, 146.88, 141.56, 137.42, 135.34, 131.42, 130.41, 129.51, 127.73, 124.03, 121.88, 120.13, 106.39, 104.82, 100.88, 99.02, 74.23, 66.59, 65.60, 60.81, 55.10, 50.89, 38.42, 36.45, 32.06, 31.58, 30.78, 30.26, 29.37, 27.99, 27.45, 26.41, 19.81, 19.01, 8.15. Yield: 48 mg, 40%. HRMS (ESI) m/z calcd for C_54_H_58_N_9_O_12_^+^ [M^+^]: 1024.4205; found 1024.4219.

#### General procedure for the synthesis of compounds 17 and 18

*1-(4-((S)-2-((S)-2-(6-(2,5-dioxo-2,5-dihydro-1H-pyrrol-1-yl)hexanamido)-3-methylbutanamido)propanamido)benzyl)-4-((7S,8S)-7-ethyl-7-hydroxy-8-methoxy-9,12-dioxo-8,9,12,14-tetrahydro-7H,11H-[1,3]dioxolo[4,5-g]oxepino[3’,4’:6,7]indolizino[1,2-b]quinolin-15-yl)pyridin-1-ium* **17**. Compound **7** (160 mg, 0.29 mmol) and **WL-14** (60 mg, 0.12 mmol) were dispersed in 2 mL of anhydrous DMF and reacted at 45°C for 24 h. After the reaction was completed, DMF was removed under reduced pressure to obtain the crude product, which was separated by semi-preparative HPLC (ACN: H_2_O = 9:11) to give a yellow solid. ^1^H NMR (400 MHz, Methanol-*d*_4_) δ 9.87 (s, 1H), 9.30 (s, 2H), 8.52–8.27 (m, 3H), 7.99 (d, J = 7.4 Hz, 1H), 7.79 (d, J = 7.8 Hz, 2H), 7.61 (d, J = 8.2 Hz, 2H), 7.50 (d, J = 9.1 Hz, 1H), 7.37 (d, J = 12.7 Hz, 1H), 6.93 (d, J = 8.0 Hz, 1H), 6.75 (s, 2H), 6.20 (d, J = 6.9 Hz, 2H), 5.95 (s, 2H), 5.66 (d, J = 15.3 Hz, 1H), 5.38 (d, J = 14.9 Hz, 1H), 4.95 (d, J = 9.5 Hz, 2H), 4.46 (t, J = 6.8 Hz, 1H), 4.11 (dt, J = 7.2, 3.6 Hz, 1H), 3.55 (s, 3H), 3.44 (t, J = 6.9 Hz, 2H), 2.29 (q, J = 7.0 Hz, 3H), 2.07 (dt, J = 13.8, 6.9 Hz, 1H), 1.92 (dd, J = 14.3, 7.5 Hz, 1H), 1.58 (dp, J = 30.3, 7.3 Hz, 4H), 1.45 (d, J = 7.1 Hz, 3H), 1.34–1.22 (m, 3H), 0.98 (t, J = 6.3 Hz, 6H), 0.81 (t, J = 7.5 Hz, 3H). ^13^C NMR (100 MHz, Methanol- *d*_4_) δ 172.60, 172.30, 150.72, 149.20, 146.86, 135.33, 131.45, 130.41, 121.86, 106.30, 102.04, 100.86, 65.58, 62.41, 60.82, 59.38, 51.24, 51.17, 38.44, 36.40, 34.14, 31.59, 29.40, 27.48, 26.35, 19.74, 18.93, 17.86, 9.02. Yield: 60 mg, 46%. HRMS (ESI) m/z calcd for C_53_H_56_N_7_O_12_^+^ [M^+^]: 982.3987; found 982.3960.

*1-(4-((S)-2-((S)-2-(6-(2,5-dioxo-2,5-dihydro-1H-pyrrol-1-yl)hexanamido)-3-methylbutanamido)-5-ureidopentanamido)benzyl)-4-((7S,8S)-7-ethyl-7-hydroxy-8-methoxy-9,12-dioxo-8,9,12,14-tetrahydro-7H,11H-[1,3]dioxolo[4,5-g]oxepino[3’,4’:6,7]indolizino[1,2-b]quinolin-15-yl)pyridin-1-ium* **18**. According to the synthesis of compound **17**, yellow solid. ^1^H NMR (400 MHz, Methanol- *d*_4_) δ 9.32 (s, 2H), 8.56–8.25 (m, 3H), 7.77 (d, J = 8.4 Hz, 2H), 7.60 (d, J = 8.4 Hz, 2H), 7.47 (s, 1H), 7.33 (s, 1H), 6.90 (s, 1H), 6.75 (s, 2H), 6.19 (d, J = 9.1 Hz, 2H), 5.96 (s, 2H), 5.64 (d, J = 15.3 Hz, 1H), 5.35 (d, J = 15.0 Hz, 1H), 4.87 (t, J = 9.5 Hz, 3H), 4.49 (dd, J = 8.9, 4.7 Hz, 1H), 4.14 (d, J = 7.2 Hz, 1H), 3.54 (s, 3H), 3.31 (s, 4H), 3.15 (ddt, J = 37.1, 13.4, 6.7 Hz, 2H), 2.30 (dt, J = 14.9, 7.4 Hz, 3H), 2.07 (h, J = 6.8 Hz, 1H), 1.90 (dq, J = 12.9, 6.9, 5.6 Hz, 2H), 1.76 (dq, J = 9.5, 4.8, 4.3 Hz, 1H), 1.57 (dp, J = 29.8, 7.3 Hz, 7H), 1.33–1.23 (m, 3H), 0.96 (d, J = 6.5 Hz, 6H), 0.80 (t, J = 7.4 Hz, 3H). ^13^C NMR (100 MHz, Methanol- *d*_4_) δ 176.50, 174.19, 172.61, 172.29, 162.35, 160.75, 155.90, 153.82, 152.13, 150.63, 149.15, 146.88, 145.96, 141.50, 137.38, 135.34, 131.46, 130.42, 129.53, 127.71, 124.54, 123.99, 121.84, 106.27, 104.80, 101.99, 100.82, 78.51, 65.61, 62.44, 60.87, 59.40, 55.11, 51.14, 38.44, 36.47, 34.15, 31.58, 30.24, 29.36, 27.44, 26.40, 19.84, 19.03, 9.02. Yield: 63 mg, 50%. HRMS (ESI) m/z calcd for C_56_H_62_N_9_O_13_^+^ [M^+^]: 1068.4467; found 1068.4446.

### Stability analysis

The compounds to be tested were dissolved in DMSO to prepare a mother liquor at a concentration of 5 mM, and the system was diluted to 50 μM with 0.01 M PBS buffer (pH = 7.4) or plasma. The system was then shaken at 37°C and 200 μL was sampled on days 0, 1, 3, 5 and 7, respectively, and the amount of free drug in the system was determined by an external standard quantitative method of HPLC. The drug release rate was calculated to evaluate the stability of the compounds *in vitro* under simulated physiological conditions (pH = 7.4) and in plasma.

### Cathepsin B release assay

Dissolve 10 units of cathepsin B in 1 mL of ABS buffer solution (pH = 5.0) containing 1 mM EDTA to prepare cathepsin B mother liquor and store at -80°C. Dissolve 5 mg EDTA and 5 mg DTT in 1 mL of ABS buffer solution (pH = 5.0) to prepare the enzyme activation solution. Since the linker-drug complexes to be tested contain a highly reactive maleimide that may affect the detection of the enzymatic release reaction, we referred to the relevant literature @ and pretreated it by adding 3 equivalents of N-acetylcysteine to bind to the linker-drug complexes. The procedure was as follows: 20 μL of a 5 mM solution of the linker-drug complex was reacted with 20 μL of 15 mM N-acetylcysteine aqueous solution in 1.6 mL of ABS buffer solution (pH = 5.0) by shaking for 30 min. Subsequently, 240 μL of enzyme activation solution was taken at 37°C for enzyme activation of 120 μL of cathepsin B mother liquor for 15 min. Finally, the activated cathepsin B solution was co-incubated with the pretreated linker-drug complex at 37°C. At different time points, 40 μL was sampled and 160 μL of cold acetonitrile was added. After centrifugation (8000 rpm) at 4°C for 15 min, the supernatant was removed and the amount of drug released from the system (n = 3) was determined by an external standard quantification method of HPLC to determine whether the linker-drug complex could be cleaved by cathepsin B and effectively release the effector molecules *in vitro*.

### Preparation and analysis of antibody drug conjugates

The novel ADCs involved in this paper were prepared on a small scale (5 mg), and the general method is as follows. Under sterile conditions, a pH = 7 buffer solution (50 mM PB, 50 mM NaCl, 2 mM EDTA) was first configured and filtered through a 0.22 μm Steriflip filter. Commercially available trastuzumab was exchanged into the configured buffer solution using an AminconUltra-4 (30KDaMWCO) centrifuge ultrafiltration tube, centrifuged twice at 5000 rpm for 20 min. The monoclonal antibody was diluted with the buffer solution, and the protein concentration was measured and controlled at about 5.0 mg/ml by UV spectrophotometer. The dosage of monoclonal antibody (the molecular weight of trastuzumab is about 148312) used was calculated according to the dosage of linker-drug complex. Since the DAR value of the target ADC is expected to be 8, it is necessary to ensure that the equivalent amount of linker-drug complex is about 8 ~ 10 times than that of the monoclonal antibody. Using TCEP as reducing agent, 8 ~ 10 equivalents of TCEP was dissolved in DMSO and maintain its concentration at 10 mg/mL. Then a calculated amount of buffer solution containing the monoclonal antibody was added to an autoclaved Argos reaction tube, slowly added DMSO solution of TCEP and activate the monoclonal antibodies for 1 h at 20 ~ 25°C. Dissolve the linker-drug complex in DMSO (the amount of DMSO should not exceed 10% of the total reaction solution volume) and added to the reaction system, and the reaction temperature is maintained at 20 ~ 25°C for approximately 30 min. After the reaction, the solution was changed with Amincon Ultra-4 (30 KDa MWCO) centrifugal ultrafiltration tubes at 6000 rpm, and the solution was changed 4 ~ 5 times to ensure that the free small molecules and salts in the system were filtered out. After that, the ADC was diluted with buffer solution, and the protein concentration was measured and controlled by UV spectrophotometer at about 5.0 mg/mL. The prepared ADC mother liquor was dispensed and transferred to lyophilization tubes and stored at -80°C. The weighted average DAR value and SEC value assay methods are provided in supplementary information.

### *In vitro* antitumor activity analysis

Cells were distributed into 96-well white round-bottom plates, with each well receiving 1000 cells in RPMI-1640 medium supplemented with 10% FBS. Following a 24-hour incubation period, diluted compounds were introduced to the wells. After 144 hours, a CellTiter-Glo luminescent cell viability assay (Promega, Madison, WI, USA) was performed to assess cell viability. The luminescent readings were normalized as percentages relative to untreated cells, and the IC50 values for each compound were determined.

## Supporting information

S1 File (DOCX)
