# Supplementary material for: Synthesis and biological evaluation of novel quaternary ammonium antibody drug conjugates based on camptothecin derivatives
Source: PLoS One. 2023 Dec 19;18(12):e0292871. doi: 10.1371/journal.pone.0292871 (PMC10729962; doi:10.1371/journal.pone.0292871)
Supplement: S1 File — (DOCX) [file pone.0292871.s001.docx]

**Synthesis and biological evaluation of novel quaternary ammonium antibody drug conjugates based on camptothecin derivatives**

Yifan Zhang ^a,b^#, Mengyuan Ding ^e^#, Lei Wang ^e^, Sicheng Yin ^b^, Liang Zhang ^b^, Xuemei Cao ^b^, Zhiyang Chen ^e^, Weinan Li ^b^, Qingsong Guo ^b^, Shulei Zhu ^c,d,e,*^, Wei Lu ^d,e,*^, Tong Yang ^b,*^

^a^ State Key Laboratory of Genetic Engineering, Department of Biochemistry, School of Life Sciences, Fudan University, Shanghai, 200438, China

^b^ R&D Department of Genetic Engineering, Shanghai Fudan-Zhangjiang Bio-Pharmaceutical Co., Ltd., Shanghai, China

^c^ Innovation Center for AI and Drug Discovery, East China Normal University, 3663 North Zhongshan Road, Shanghai, 200062, PR China.

^d^ Shanghai Key Laboratory of Intelligent Drug Design and Manufacturing, East China Normal University, 3663 North Zhongshan Road, Shanghai, 200062, PR China.

^e^ Shanghai Engineering Research Center of Molecular Therapeutics and New Drug Development, School of Chemistry and Molecular Engineering, East China Normal University, 3663 North Zhongshan Road, Shanghai, 200062, PR China.

*** Corresponding authors**

[tyang@fd-zj.com](mailto:tyang@fd-zj.com) (Tong Yang)

[wlu@chem.ecnu.edu.cn](mailto:wlu@chem.ecnu.edu.cn) (Wei Lu)

[slzhu@chem.ecnu.edu.cn](mailto:slzhu@chem.ecnu.edu.cn) (Shulei Zhu)

# These authors contributed equally.

**Supporting Information**

**S1 Scheme. Reagents and conditions: (a) THF, rt, 12 h, 50%; (b) p-amino benzyl alcohol, EEDQ, DCM/MeOH, rt, 12 h, 80%; (c) diethyl amine, THF, rt, 4 h, 70%; (d) THF, rt, 12 h, 80%.**

**S2 Scheme. Reagents and conditions: (a) THF, rt, 12 h, 42%; (b) p-amino benzyl alcohol, EEDQ, DCM/MeOH, rt, 12 h, 83%; (c) diethyl amine, THF, rt, 4 h, 80%; (d) THF, rt, 12 h, 80%.**

**The assay of weighted average DAR value**

We used reverse high-performance liquid chromatography (RP-HPLC) to determine the weighted average DAR of the prepared ADCs. The specific process is as follows:

Preprocessing of ADC samples: ADC was diluted with ultrapure water to a protein concentration of 1 mg/mL. 60 µL of ADC sample was taken, 15 µL aqueous solution containing 100 mM DTT was added and reacted at 37 ℃ for 30 min after thoroughly mixed. After the reaction was completed, the system was centrifuged at 15000 rpm for 10 min and directly inject the sample for analysis.

Selection of liquid phase method: Mobile phase A: ACN + 0.1% TFA, Phase B: H_2_O + 0.1% TFA. Due to the differences in physical and chemical properties of linker-drug complexes of different ADCs, the peak positions of each light and heavy chain component in the HPLC analysis are also different. Therefore, we used two liquid phase methods according to practical needs during the analysis. Method one: Elution gradient for phase A: 0 ~ 3.0 min: 15% ~ 29%, 3.0 ~ 6.0 min: 29% ~ 40%, 6.0 ~ 20.0 min: 40% ~ 55%, 20.0 ~ 20.1 min: 55% ~ 15%, 20.1 ~ 30.0 min: 15%, 30.0 min: end of the method. Method two: 0 ~ 3.0 min: 27% ~ 27%, 3.0 ~ 25.0 min: 27% ~ 49%, 25.0 ~ 26.0 min: 49% ~ 55%, 26.0 ~ 31.0 min: 95%, 31.0 ~ 35.0 min: 95% ~ 27%, 35.0 min: end of the method. Column type: Agilent PLRP-S, 1000Å, 8 µm, 50*2.1 mm, PN PL1912-1802. Flow rate: 0.25 mL/min, the instrument: Waters Acquity Arc. Column temperature chamber: 70 °C.

**The assay of SEC value**

The chromatographic conditions were as follows: test instrument: Waters Acquity Arc. Column type: TOSOH, TSKgel G3000SW_XL_, 7.8*300 mm. Mobile phase A: 100 mM PB + 200 mM Arg-HCl + 5% IPA, elution gradient of phase A: 0 ~ 20 min: 100%, 20 min: end of the method. Flow rate: 0.6 mL/min. Column temperature chamber: 30°C.


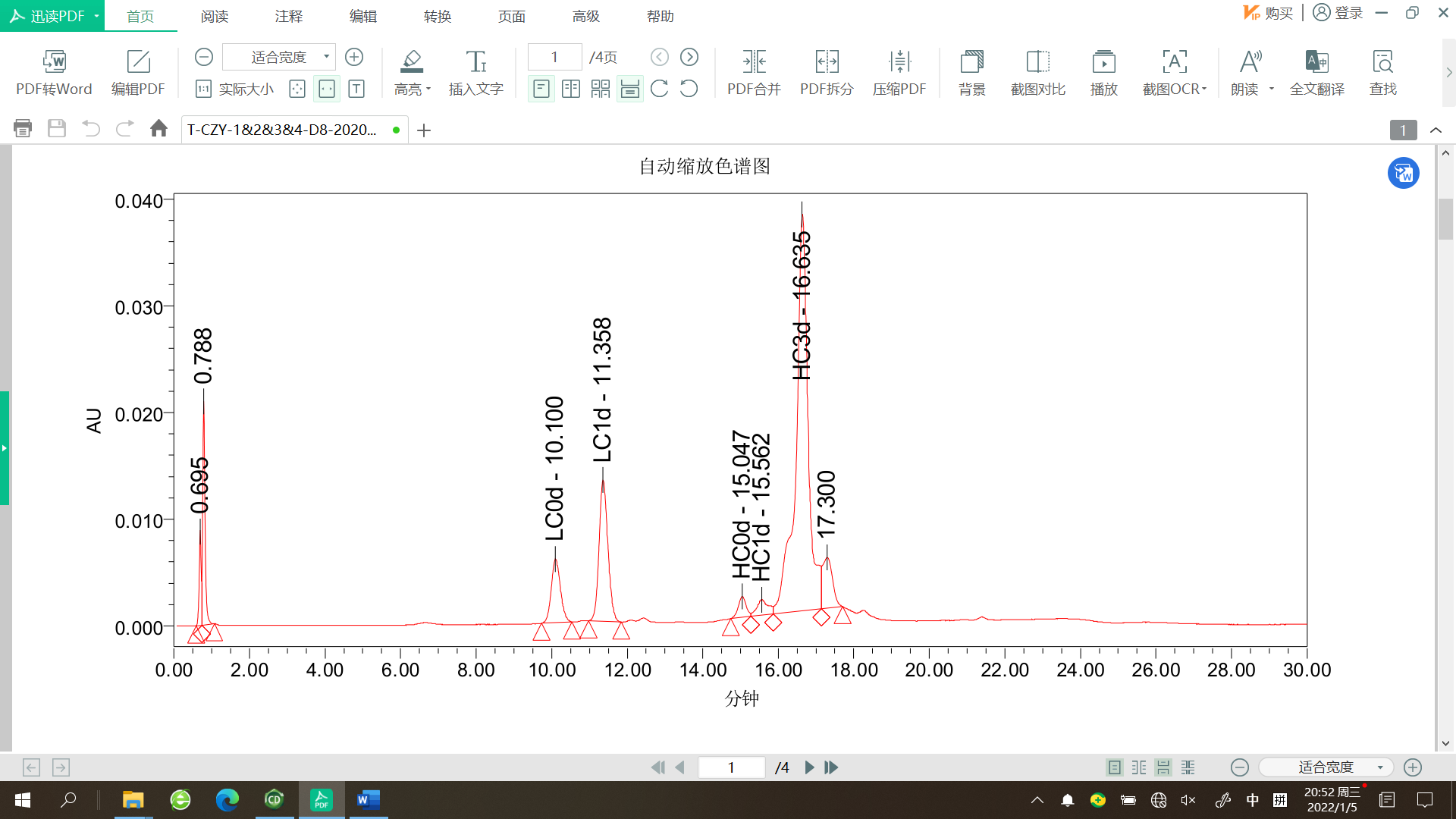


**S1 Fig. The RP-HPLC liquid phase spectrum of HER2-14**

**S1 Table. Weighted average DAR for HER2-14**

| Name of the peak ^a^ | t _R_ (min) | | Peak area (µV*sec) | Percentage of peak area ^b^/% | Weighted peak area ^c^/% |
| --- | --- | --- | --- | --- | --- |
| (LC-0d) | 10.100 | | 100339 | 22.06 | 0 |
| (LC-1d) | 11.358 | | 215004 | 68.18 | 68.18 |
| (HC-0d) | 15.047 | | 27941 | 3.07 | 0 |
| (HC-1d) | 15.562 | | 848746 | 3.46 | 3.46 |
| (HC-3d) | 16.635 | | 86146 | 93.46 | 280.38 |
| Weighted average DAR | |  |  |  | 7.0 |

^a^ LC represents light chain and HC represents heavy chain. 0d represents a drug load of 0, 1d represents a drug load of 1 and 3d represents a drug load of 3.

^b^ The percentage of peak area (%) represents the proportion of light or heavy chains to the respective loading fraction.

^c^ Weighted peak area = drug load × percentage of peak area.


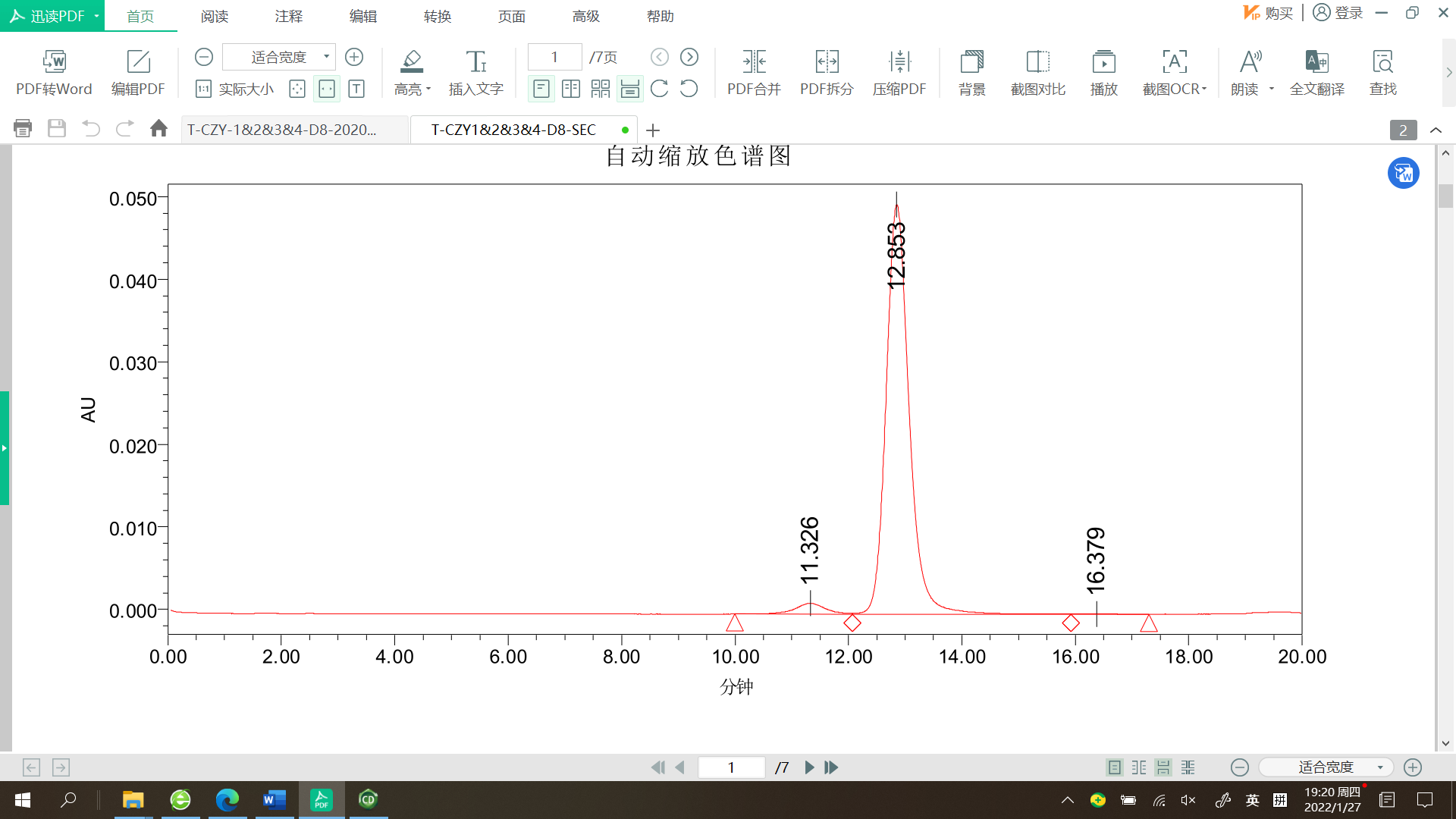


**S2 Fig. The SEC spectrum of HER2-14**


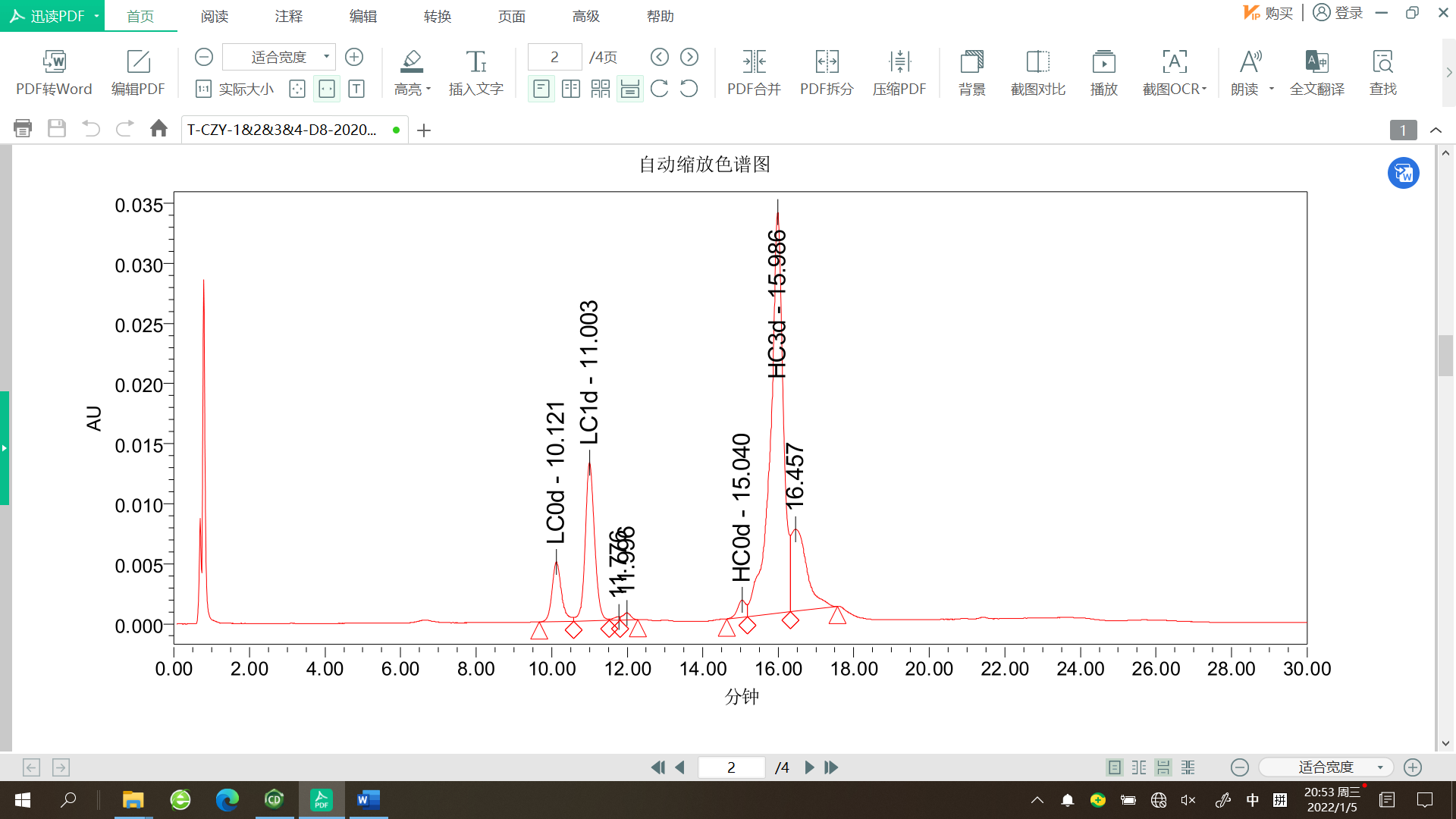


**S3 Fig. The RP-HPLC liquid phase spectrum of HER2-16**

**S2 Table. Weighted average DAR for HER2-16**

| Name of the peak ^a^ | t _R_ (min) | | Peak area (µV*sec) | Percentage of peak area ^b^/% | Weighted peak area ^c^/% |
| --- | --- | --- | --- | --- | --- |
| (LC-0d) | 10.121 | | 85935 | 27.88 | 0 |
| (LC-1d) | 11.003 | | 222484 | 72.12 | 72.12 |
| (HC-0d) | 15.040 | | 21910 | 2.68 | 0 |
| (HC-3d) | 15.985 | | 797238 | 97.32 | 291.96 |
| Weighted average DAR | |  |  |  | 7.3 |

^a^ LC represents light chain and HC represents heavy chain. 0d represents a drug load of 0, 1d represents a drug load of 1 and 3d represents a drug load of 3.

^b^ The percentage of peak area (%) represents the proportion of light or heavy chains to the respective loading fraction.

^c^ Weighted peak area = drug load × percentage of peak area.


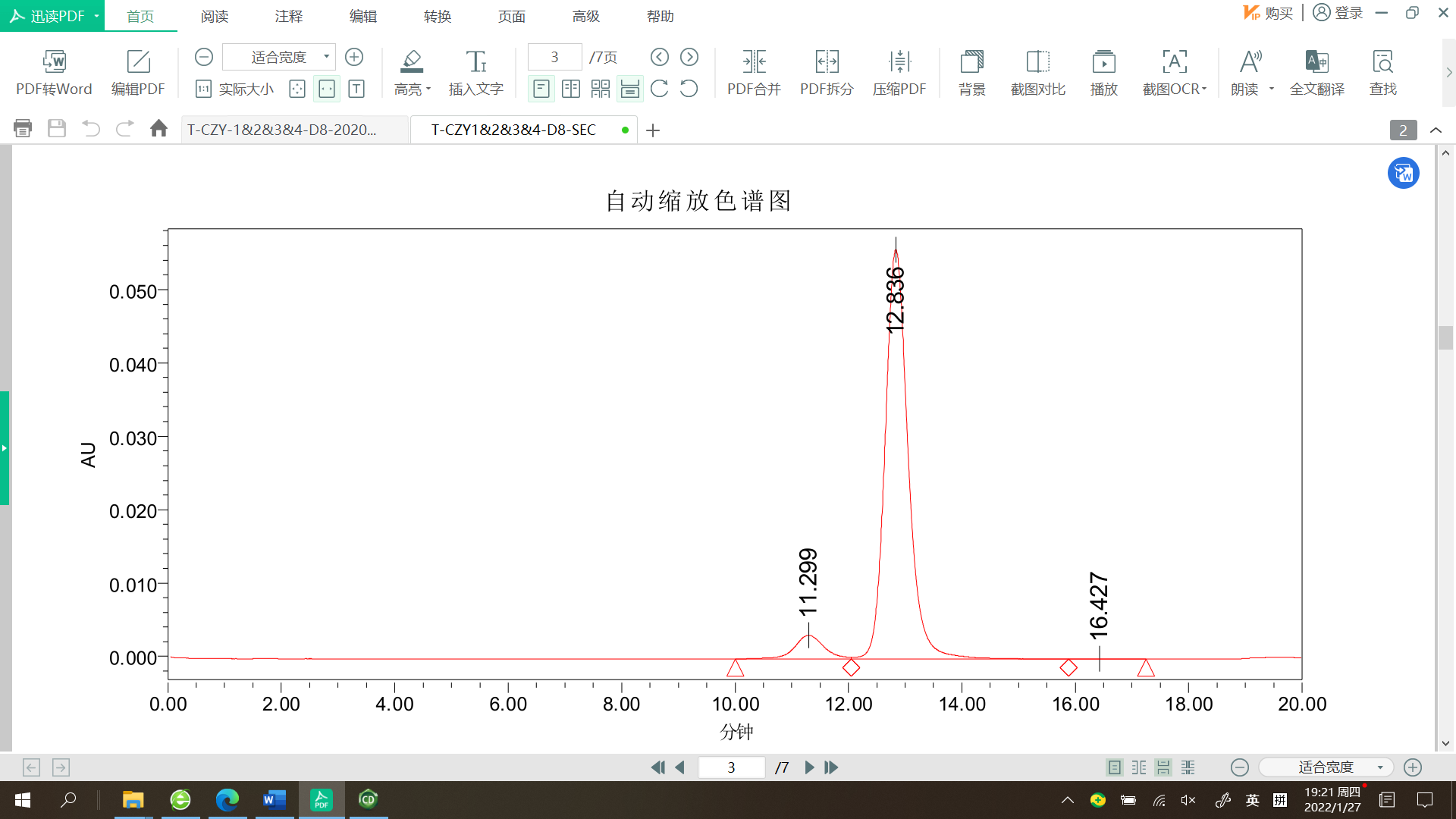


**S4 Fig. The SEC spectrum of HER2-16**

**
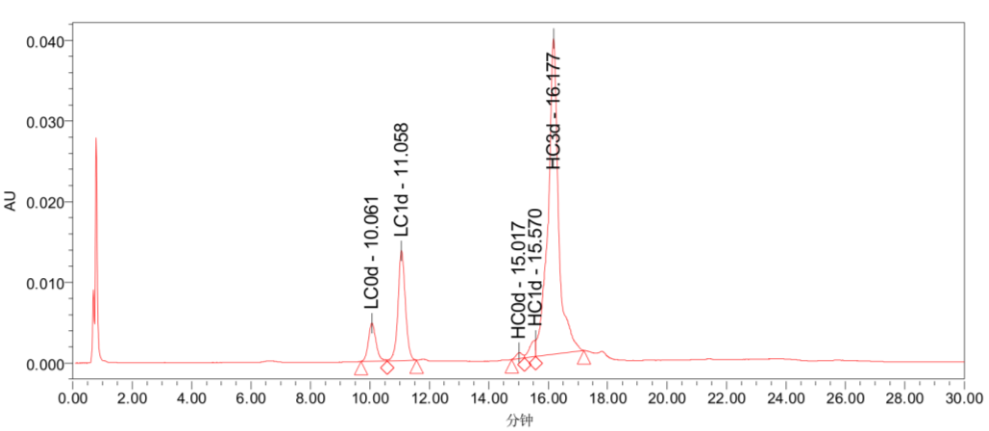
**

**S5 Fig. The RP-HPLC liquid phase spectrum of HER2-17**

**S3 Table. Weighted average DAR for HER2-17**

| Name of the peak ^a^ | t _R_ (min) | | Peak area (µV*sec) | Percentage of peak area ^b^/% | Weighted peak area ^c^/% |
| --- | --- | --- | --- | --- | --- |
| (LC-0d) | 10.061 | | 81704 | 26.15 | 0 |
| (LC-1d) | 11.058 | | 230862 | 73.85 | 73.85 |
| (HC-0d) | 15.017 | | 10192 | 1.02 | 0 |
| (HC-1d) | 15.570 | | 27176 | 2.72 | 2.72 |
| (HC-3d) | 16.177 | | 964045 | 96.26 | 288.78 |
| Weighted average DAR | |  |  |  | 7.3 |

^a^ LC represents light chain and HC represents heavy chain. 0d represents a drug load of 0, 1d represents a drug load of 1 and 3d represents a drug load of 3.

^b^ The percentage of peak area (%) represents the proportion of light or heavy chains to the respective loading fraction.

^c^ Weighted peak area = drug load × percentage of peak area.

**
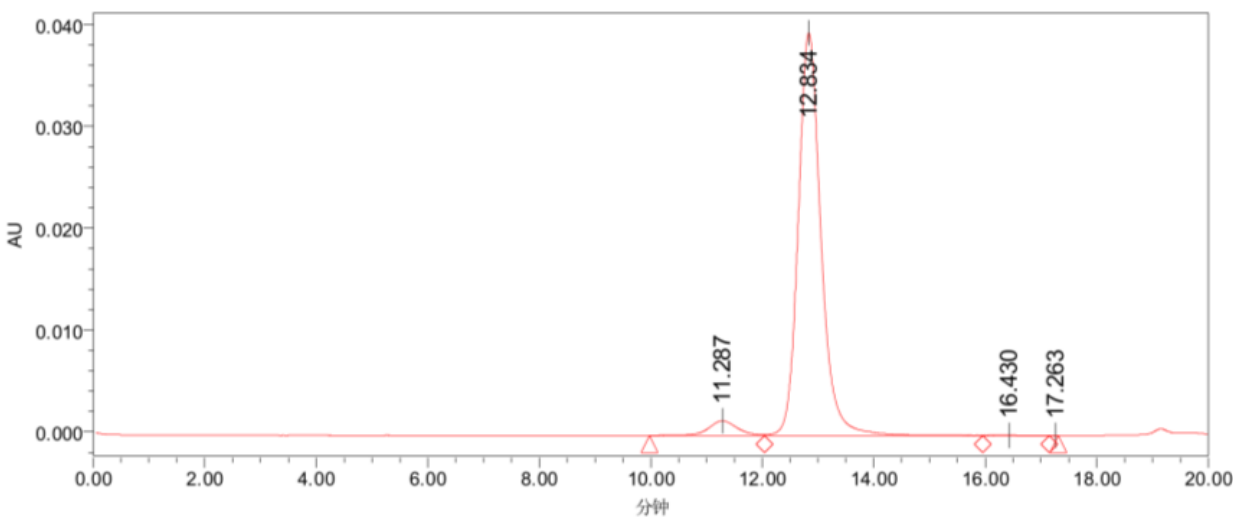
**

**S6 Fig. The SEC spectrum of HER2-17**

**
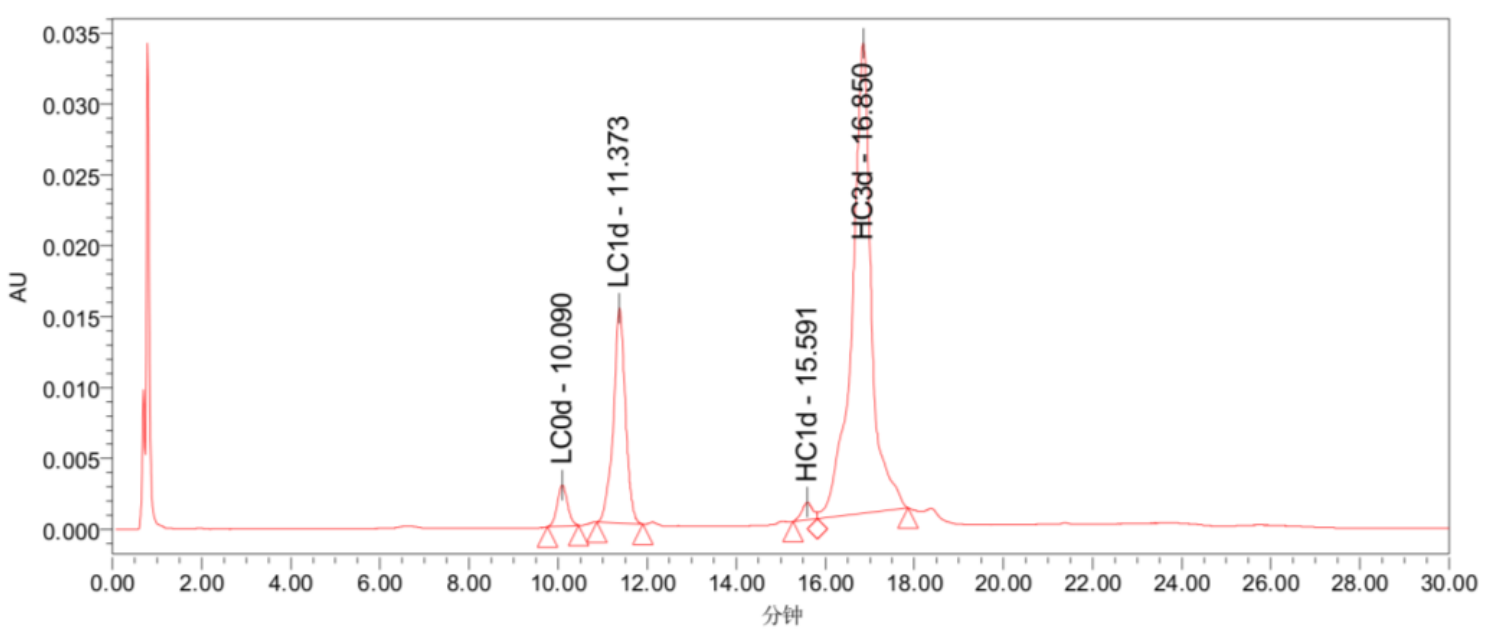
**

**S7 Fig. The RP-HPLC liquid phase spectrum of HER2-18**

**S4 Table. Weighted average DAR for HER2-18**

| Name of the peak ^a^ | t _R_ (min) | | Peak area (µV*sec) | Percentage of peak area ^b^/% | Weighted peak area ^c^/% |
| --- | --- | --- | --- | --- | --- |
| (LC-0d) | 10.090 | | 46155 | 13.99 | 0 |
| (LC-1d) | 11.373 | | 284063 | 86.01 | 86.01 |
| (HC-0d) | 15.591 | | 21236 | 2.06 | 2.06 |
| (HC-3d) | 16.850 | | 1011710 | 97.94 | 293.82 |
| Weighted average DAR | |  |  |  | 7.6 |

^a^ LC represents light chain and HC represents heavy chain. 0d represents a drug load of 0, 1d represents a drug load of 1 and 3d represents a drug load of 3.

^b^ The percentage of peak area (%) represents the proportion of light or heavy chains to the respective loading fraction.

^c^ Weighted peak area = drug load × percentage of peak area.

**
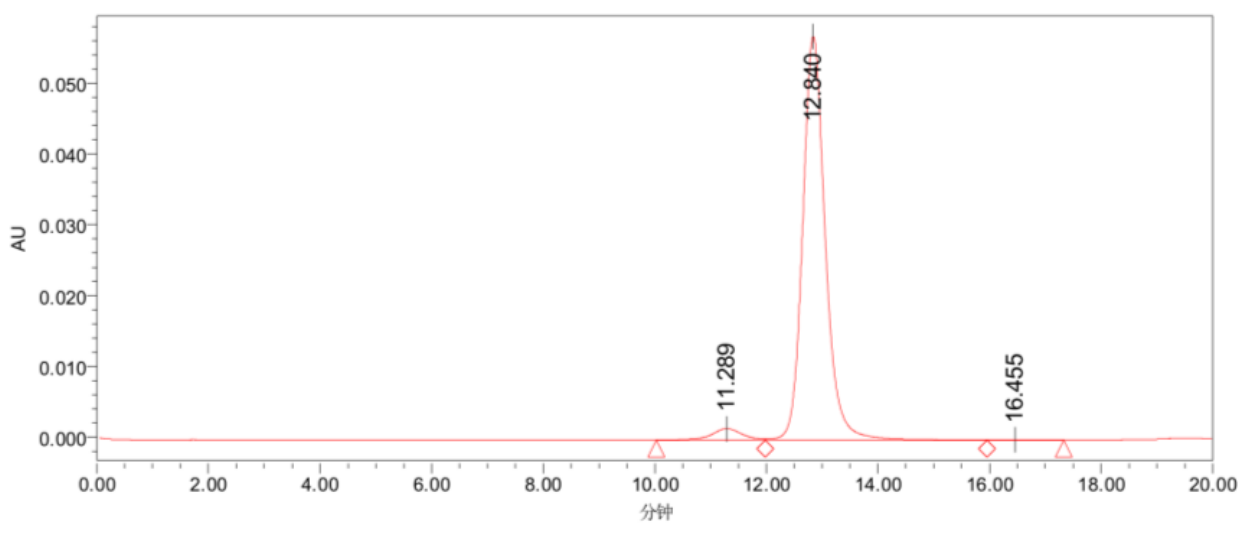
**

**S8 Fig. The SEC spectrum of HER2-18**


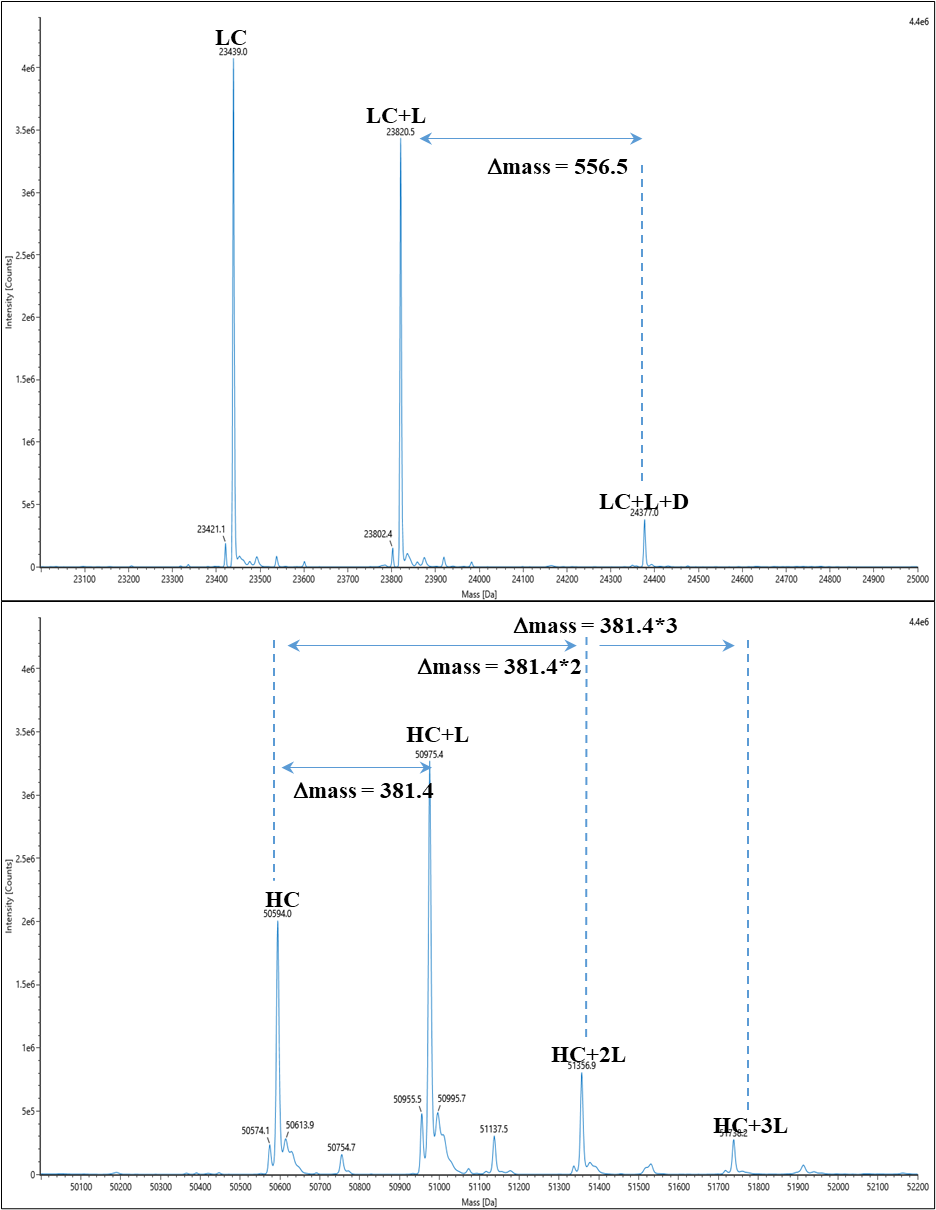


**S9 Fig. The mass spectra of HER2-14.**


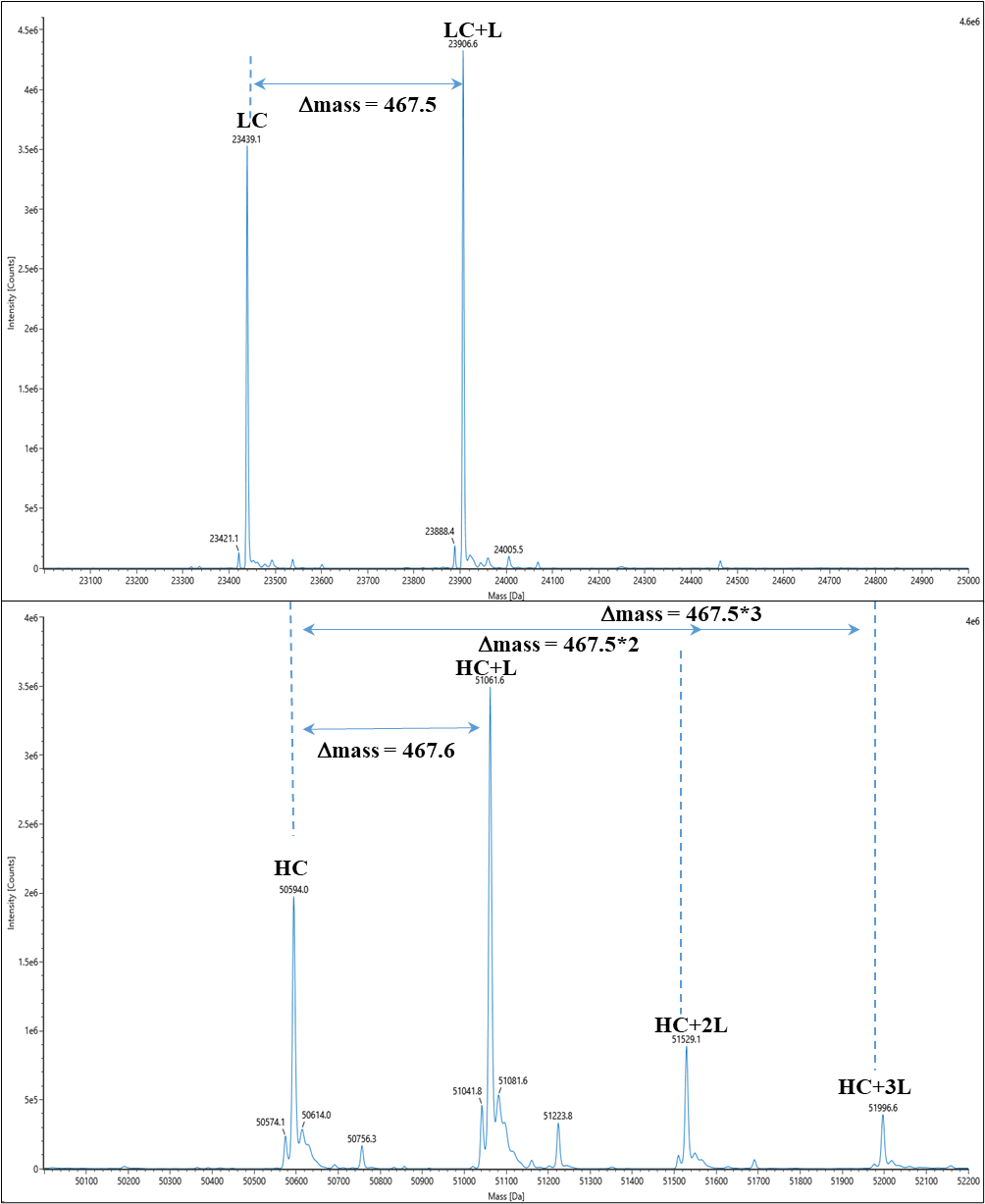


**S10 Fig. The mass spectra of HER2-16.**


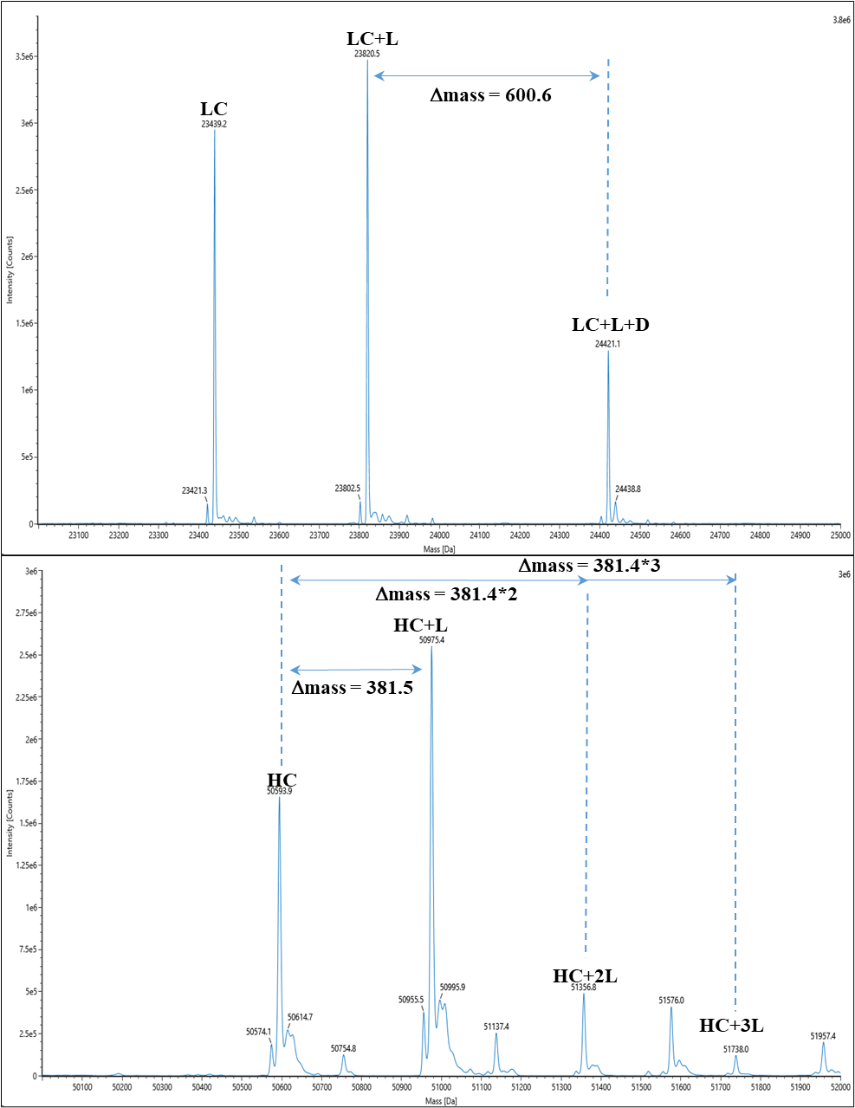


**S11 Fig. The mass spectra of HER2-17.**


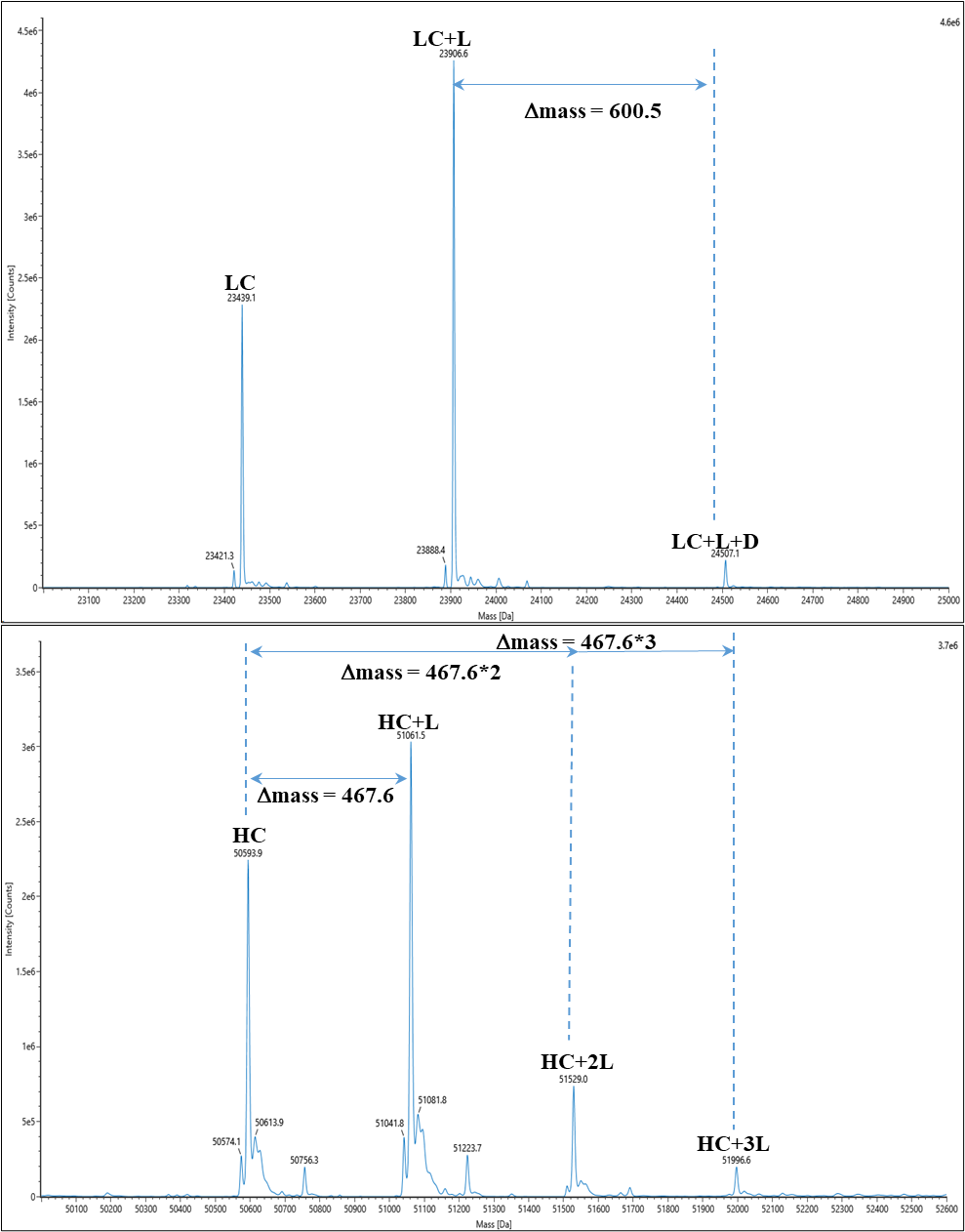


**S12 Fig. The mass spectra of HER2-18.**

**
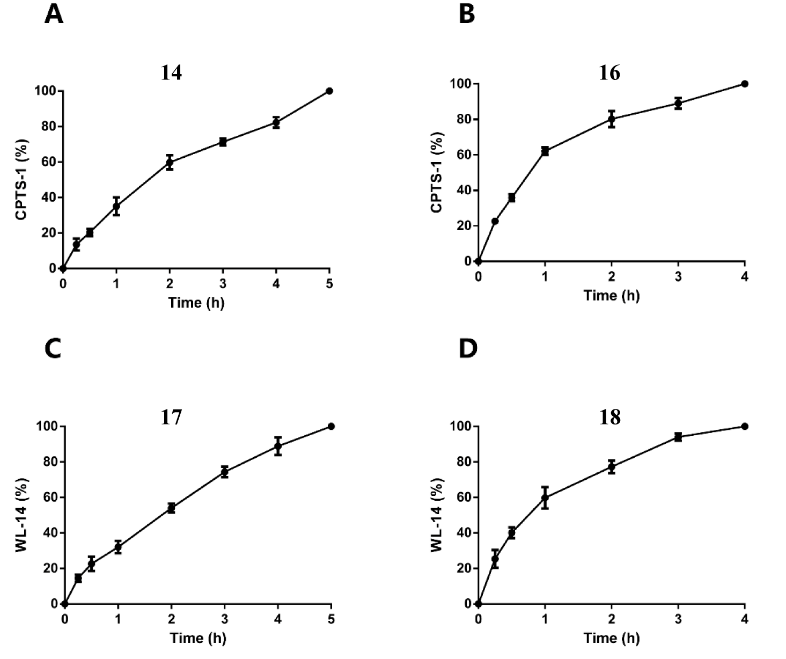
**

**S13 Fig. The release of CPTS-1 and WL-14 from** **linker-drug complexes via cathepsin B.**


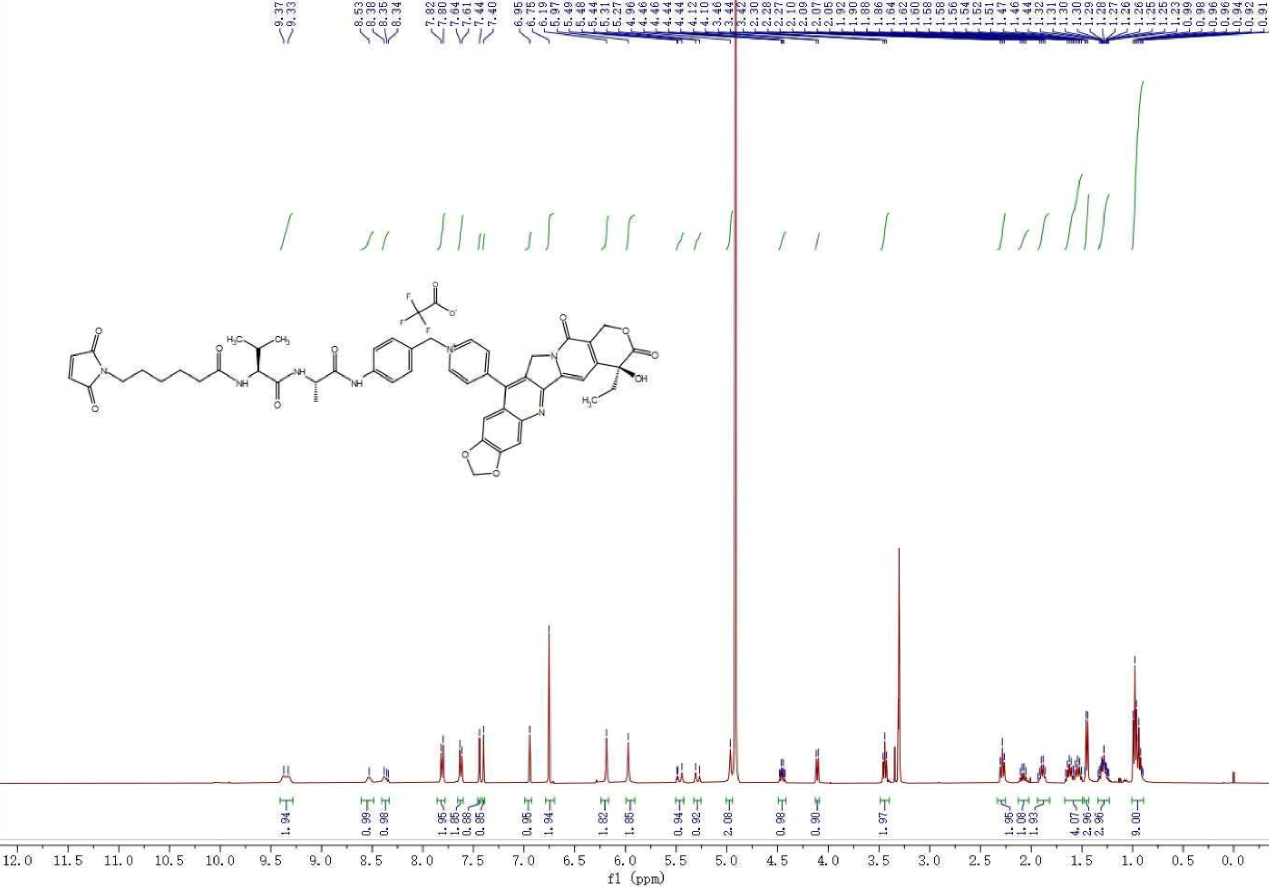


**S14 Fig. The ^1^H NMR spectrum of compound 14**

**
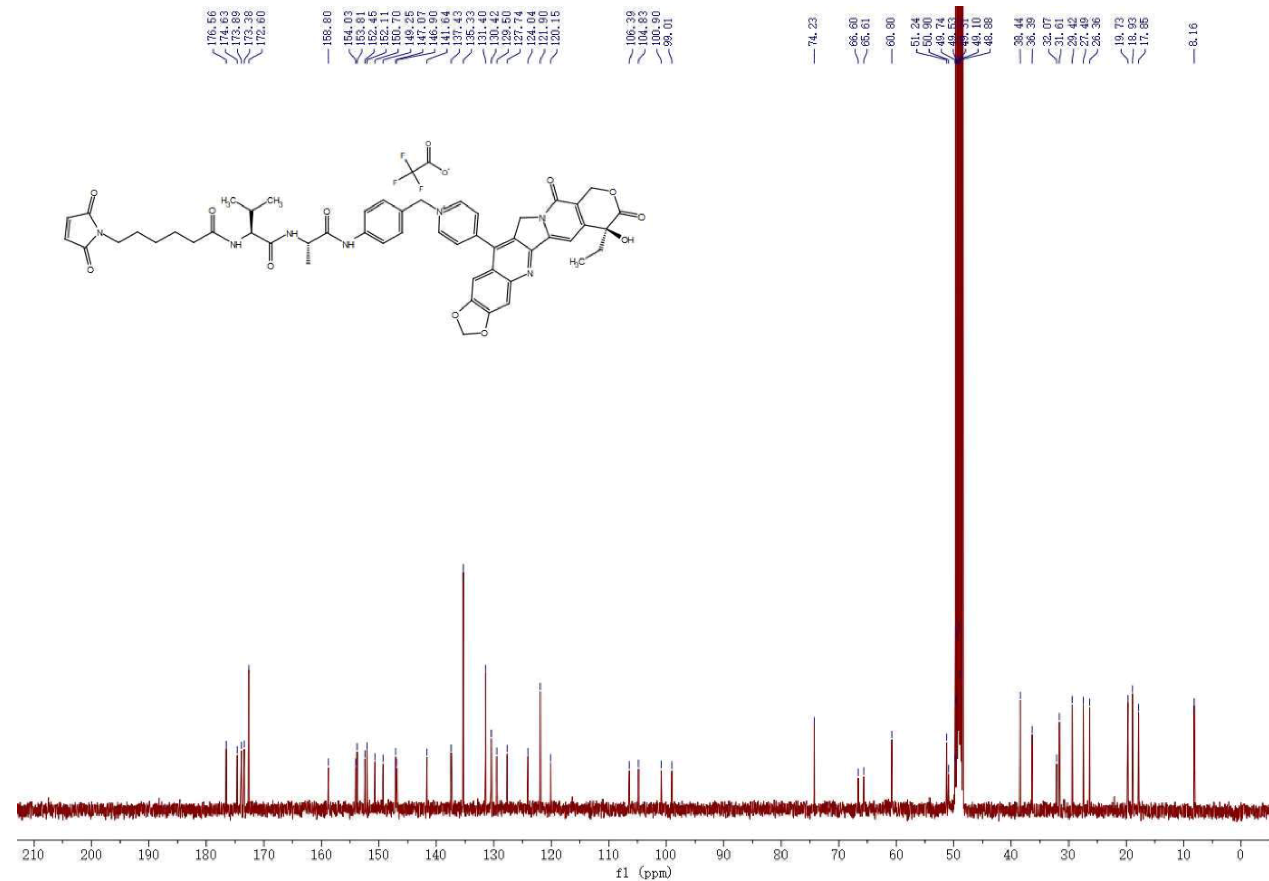
**

**S15 Fig. The ^13^C NMR spectrum of compound 14**


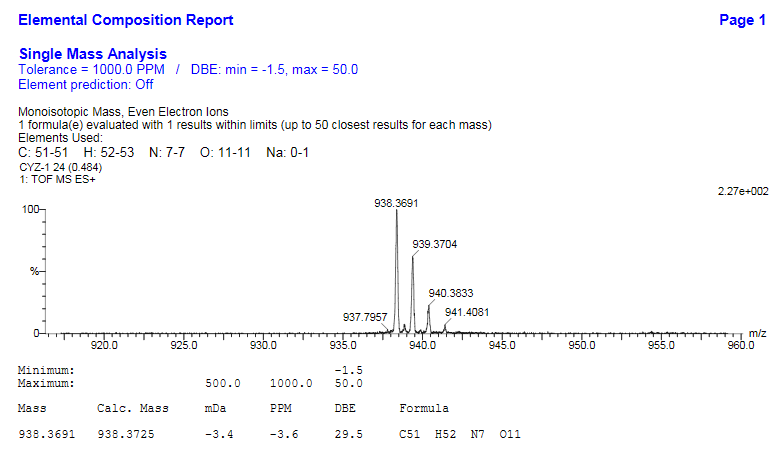


**S16 Fig. The HR-MS spectrum of compound 14**


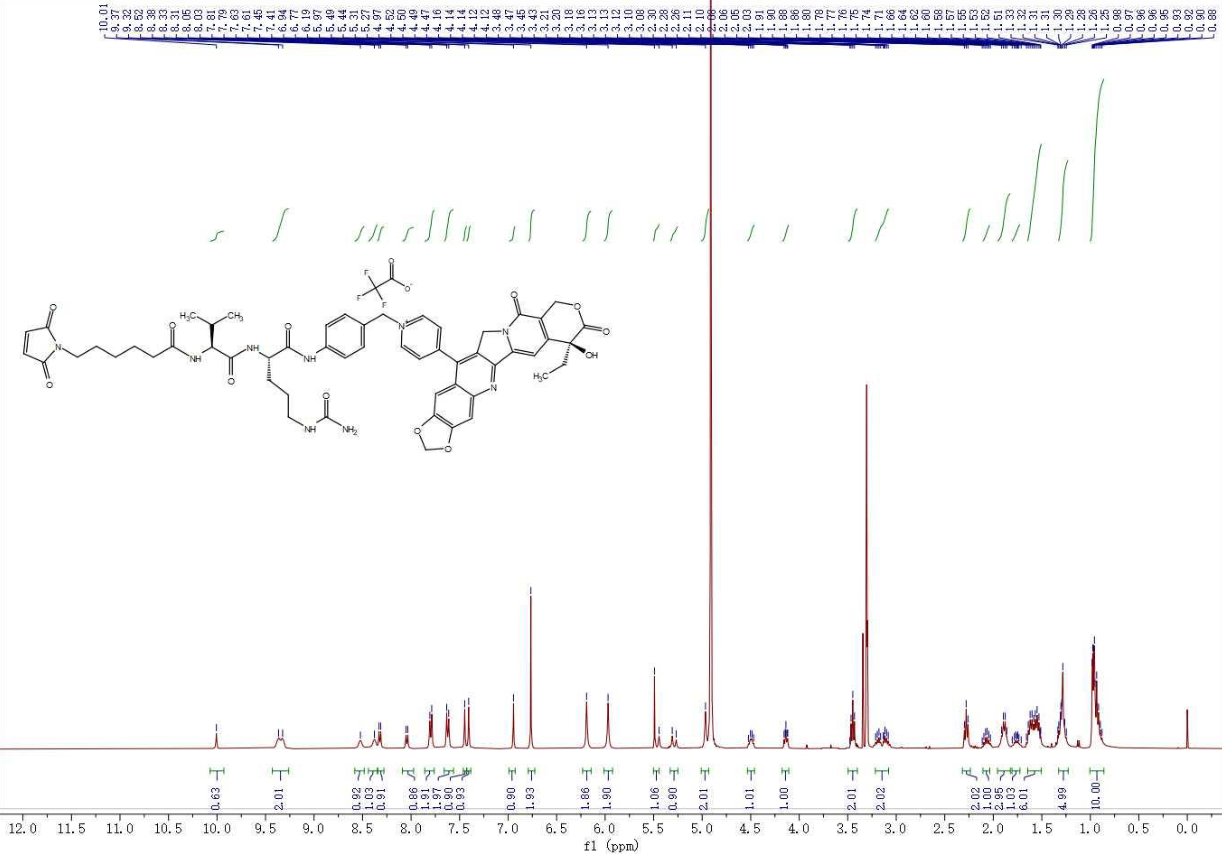


**S17 Fig. The ^1^H NMR spectrum of compound 16**

**
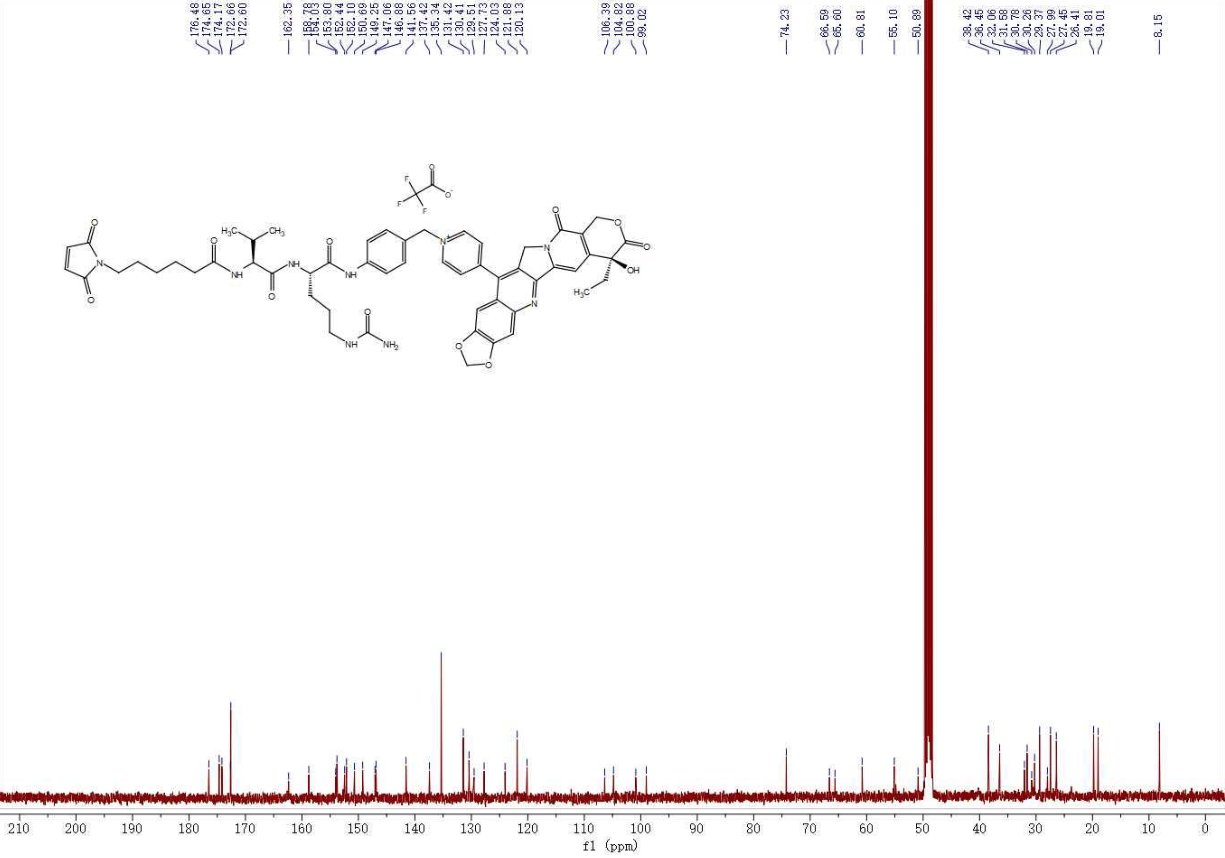
**

**S18 Fig. The ^13^C NMR spectrum of compound 16**


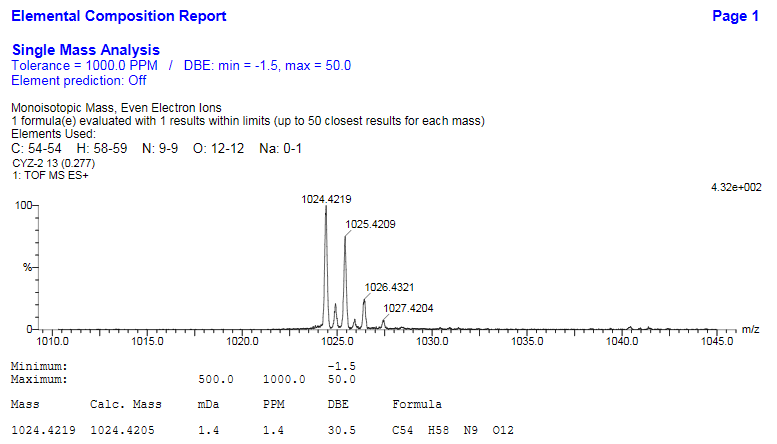


**S19 Fig. The HR-MS spectrum of compound 16**


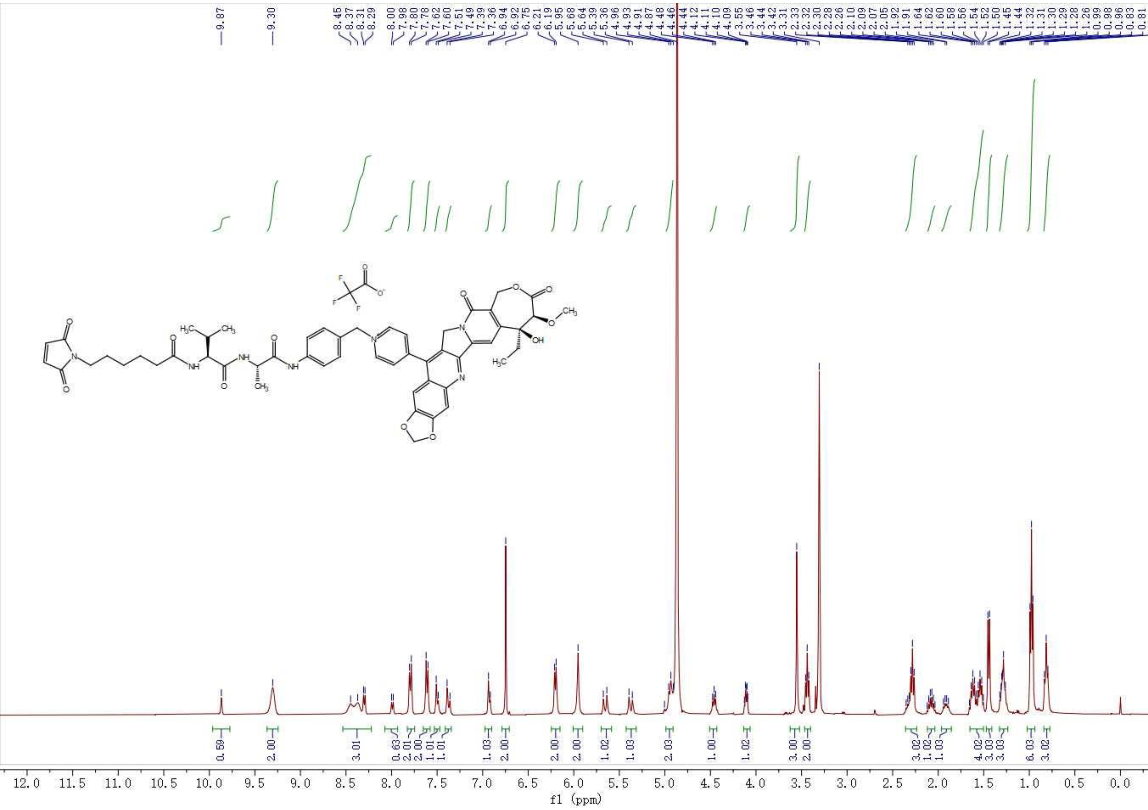


**S20 Fig. The ^1^H NMR spectrum of compound 17**


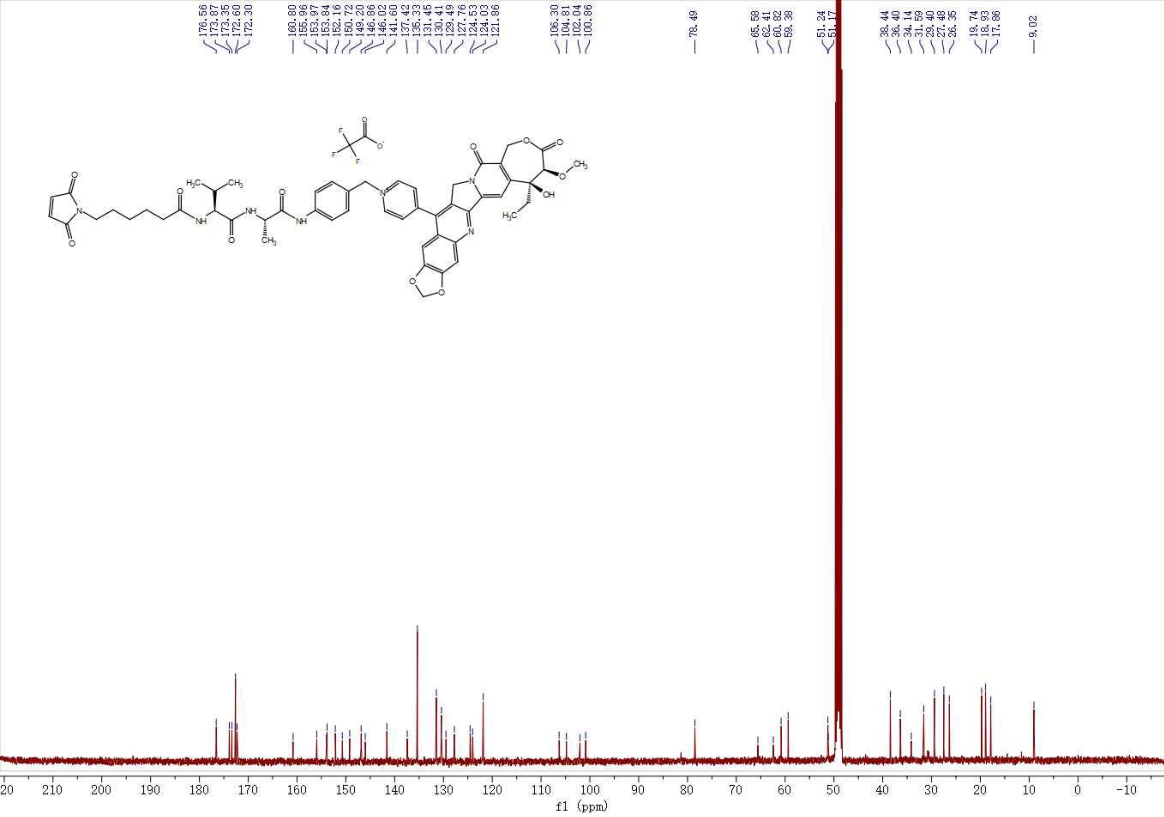


**S21 Fig. The ^13^C NMR spectrum of compound 17**


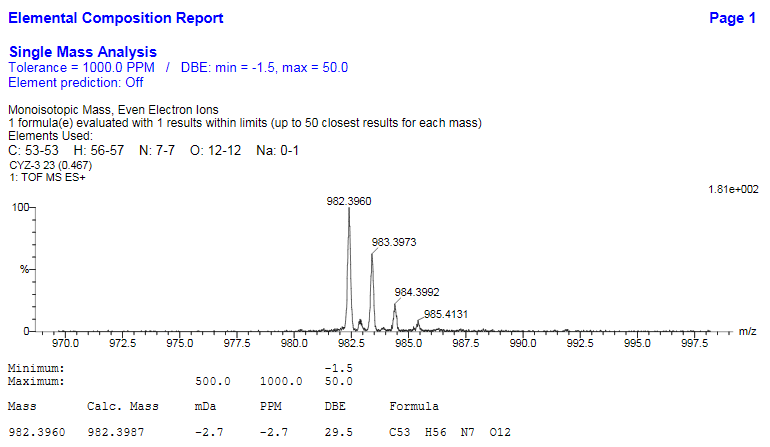


**S22 Fig. The HR-MS spectrum of compound 17**


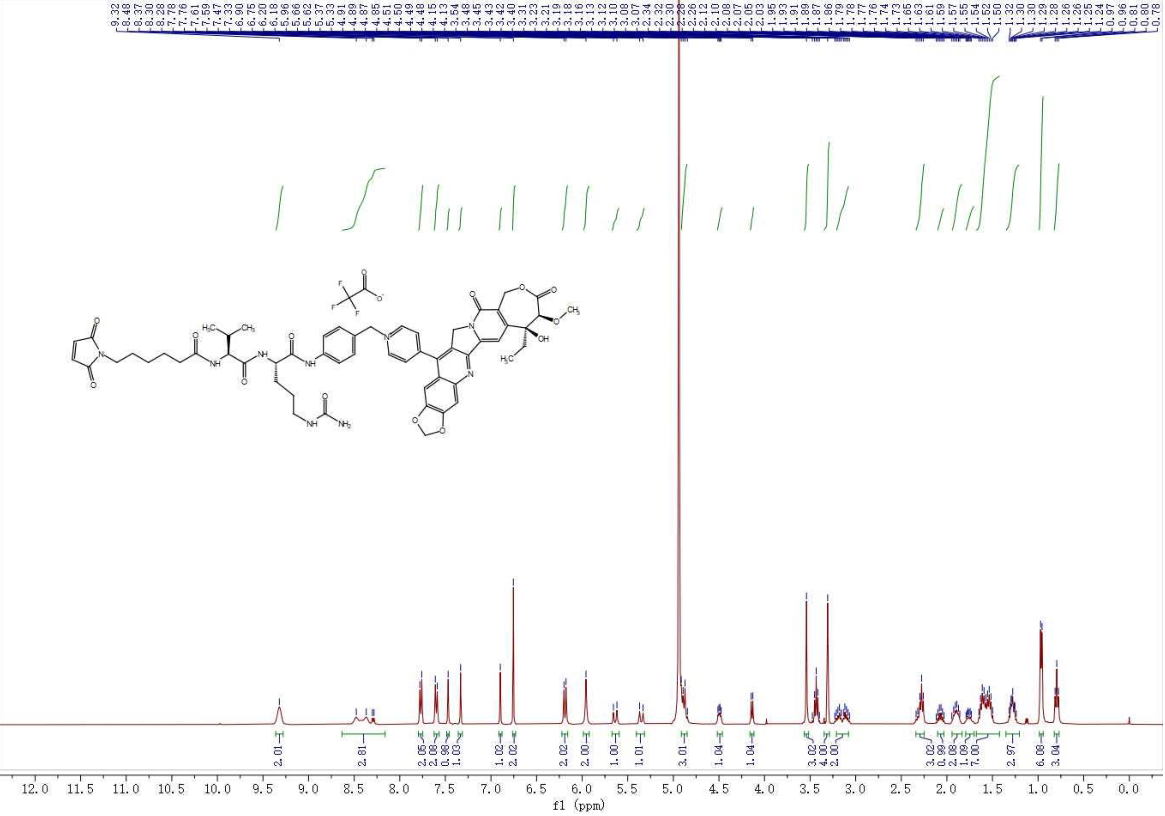


**S23 Fig. The ^1^H NMR spectrum of compound 18**


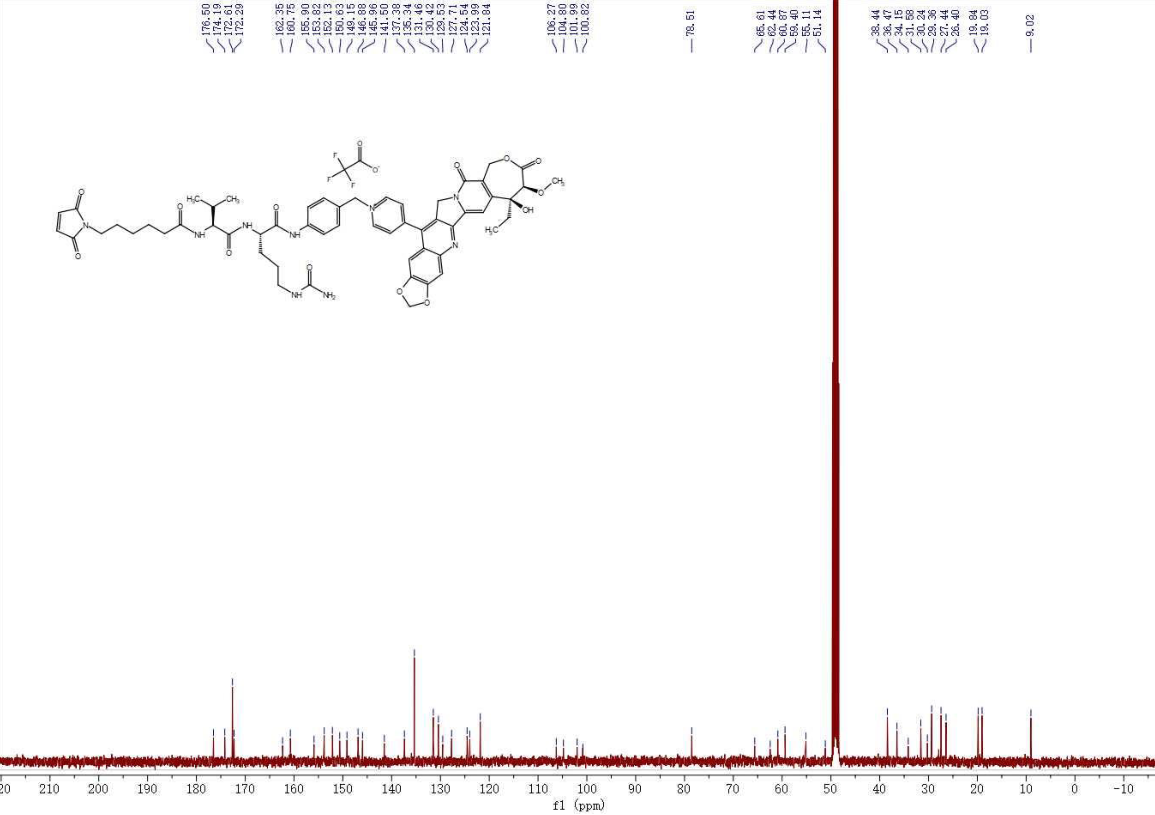
 **S24 Fig. The ^13^C NMR spectrum of compound 18**


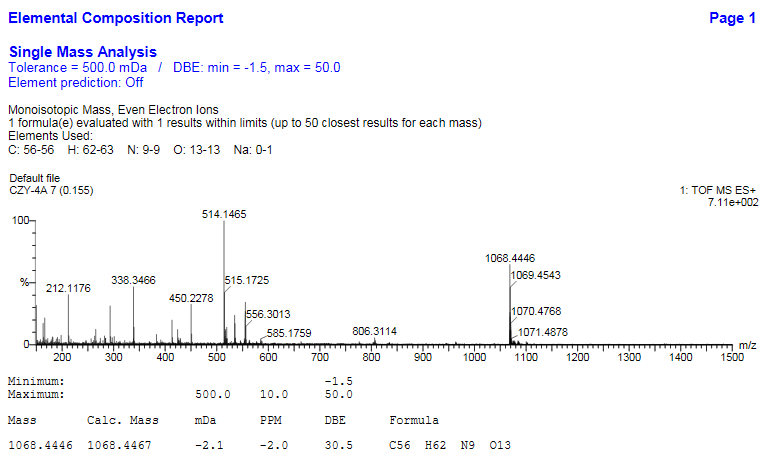


**S25 Fig. The HR-MS spectrum of compound 18**
